# Evaluation of leukocyte-platelet rich fibrin as an antibiotic slow-release biological device in the treatment of moderate periodontitis: a randomized controlled clinical trial

**DOI:** 10.1186/s12903-024-05254-x

**Published:** 2024-12-21

**Authors:** Yasmeen K. Omar, Mohy A. El Rashidy, Ghada B. Ahmed, Aliaa G. Aboulela

**Affiliations:** 1 Department of Oral Medicine, Periodontology, Oral Diagnosis and Oral Radiology, Faculty of Dentistry, Alexandria University, Champollion St, Azarita, 21527 Alexandria Egypt; 2 Department of Microbiology, Medical Research Institute, Alexandria University, Azarita, Egypt

**Keywords:** Antibacterial agents, Intrasulcular fluid, Leukocyte and platelet-rich fibrin (L-PRF), Metronidazole

## Abstract

**Background:**

Periodontitis is a chronic inflammatory disease caused by the accumulation of biofilm. Antimicrobials have been used as adjuncts to non-surgical periodontal therapy. However, systemic antibiotics often require large dosages to achieve suitable concentrations at the disease site. Leukocyte platelet-rich fibrin (L-PRF) is a promising bio-material, with antimicrobial, anti-inflammatory, and wound-healing enhancement effects. This study aimed to evaluate the efficacy of L-PRF as a locally sustained released device for metronidazole antimicrobial.

**Methods:**

Twenty-four patients with eighty periodontal pockets had moderate periodontitis with attachment loss of 3–4 mm, and probing depth ≤ 5, which was equally divided into two groups: Group (I) underwent scaling and root planing with intra-pocket application of L-PRF loaded with Metronidazole, while Group (II) was treated by scaling and root planing with intra-pocket application of L-PRF alone. Microbiological measurements were taken at baseline and after one month to analyze the relative count of *Porphyromonas gingivalis (P. gingivalis)* using real time PCR. Clinical parameters were measured at baseline and after 1, 3, and 6 months. These parameters included probing depth (PD), clinical attachment loss (CAL), plaque index (PI), modified gingival index (MGI), and bleeding index (BI).

**Results:**

Microbiological and clinical findings revealed that both treatment methods resulted in a reduction in *P. gingivalis* counts, in addition to improvements in the clinical parameters: PD reduction, CAL gain, PI reduction, BI decrease and MGI reduction compared to baseline. However, L-PRF-metronidazole group showed superior results in the studied parameters over the study period. Nonetheless, there was no statistically significant improvement. (*p* < .001).

**Conclusion:**

The intra-pocket application of both L-PRF loaded with Metronidazole and L-PRF alone contributed to the successful treatment of moderate periodontitis.

**Trial registration:**

NCT06153706 (http://www.clinical-trials.gov/); 1/12/2023, retrospective registration.

**Supplementary Information:**

The online version contains supplementary material available at 10.1186/s12903-024-05254-x.

## Introduction

Periodontitis is the most prevalent chronic inflammatory dental disease. It involves inflammation of the structures that support the teeth, mainly triggered by the buildup of complex and diverse dental plaque containing multiple microbes. This condition is initiated by Gram-negative microbial bio-films associated with teeth, which prompt the body’s immune response, leading to continuous and irreversible damage to the bone and soft tissues. Consequently, periodontal pockets form, eventually leading to tooth mobility and eventual loss [1].

Gram-negative rods like Aggregatibacter actinomycetemcomitans, Tannerella forsythia, Prevotella, Fusobacterium, and Porhyromonas gingivalis (PG) are commonly the main cause of periodontal diseases. Among the major periodontal pathogens, PG appears to be one of the main etiological agents in the beginning and development of periodontitis [2, 3]. It is an organism that thrives in low-oxygen environments. It is rare in individuals with healthy gingival tissues [4]. Anaerobic culture is the standard technique for identifying and measuring the main components of subgingival plaque and figuring out the antibiotic susceptibilities of oral infections. However, because some oral infections grow slowly and have particular conditions, this approach has drawbacks. It also takes a lot of time and has low sensitivity. As an alternative, several techniques, including PCR assays [5], immunoassays, and DNA probe assays [6], have been developed to identify PG.

It has been demonstrated that real-time PCR is an extremely quick and sensitive technique for identifying and measuring specific microbial species. Real-time PCR assays typically rely on detecting particular DNA sequences encoding the bacterial small subunit 16 S rRNA. These DNA sequences are found in multiple copies in every species of bacteria, and they are highly conserved and species-specific [7]. Porhyromonas gingivalis has been extensively identified and quantified using this technique in subgingival plaque samples.

Several strategies are used for managing periodontal disease, such as mechanical debridement, antibiotics and antiseptics to disrupt or interfere with bacterial metabolism, and altering the environment of microorganisms to affect their growth [7].

Mechanical debridement is the most widely used treatment method for managing periodontal disease, demonstrating considerable success in treating most patients. However, when used alone, it poses a higher risk of recurrence, particularly in cases with systemic co-morbidity [8]. Furthermore, it has been noted that it can be difficult to completely remove calculus and plaque by mechanical debridement in difficult-to-reach places like furcation areas and deep pockets (greater than 5 mm), which raises the risk of treatment failure [9]. SRD might not be enough, especially in hard-to-reach areas, to eradicate all bacteria from deep periodontal pockets. Antibiotics, both systemic and local, have long been used as adjuncts in the management of periodontal disease [2]. However, using systemic antibiotics frequently and for a long time can have negative effects, including low patient compliance, the development of resistant infection strains, and secondary infections [6].

Local therapy is now the recommended choice since it avoids problems related to systemic administration. The efficacy of periodontal therapy is largely dependent on the choice of an appropriate antimicrobial agent and the method of delivery. Local drug delivery (LDD) provides several advantages over systemic therapy, including reduced side effects and higher patient adherence. Studies have shown that when LDD is administered locally instead of systemically, pockets of periodontal tissue produce more therapeutic levels of antibiotics [10]. Compared to systemic therapy, several local antimicrobials are being used to treat periodontal disease, such as clindamycin (CLI), azithromycin (AZM), tetracycline (TET), metronidazole (MTZ), doxycycline (DOX), minocycline (MIN), and chlorhexidine (CHX). Currently, these antimicribials are being used in irrigations, fibres, injectables, gels, strips, microparticles, and nanoparticles for drug delivery systems [11].

Metronidazole (MTZ), a broad-spectrum antibiotic, is used to treat infections caused by protozoa and anaerobic bacteria [12]. It is the suggested course of treatment for moderate-to-severe periodontitis and is commonly used either alone or in conjunction with other antimicrobial [10, 13].

Endogenous regenerative technology, or ERT, has produced interest in the field of regeneration because it has the ability to activate patients’ dormant self-healing mechanisms and stimulate the body’s natural capacity for regeneration. One of the most widely used endogenous regenerative technology (ERT) is platelet-rich fibrin (PRF) [14]. The regenerative capability of platelet concentrates has been widely studied in the past few years. The release of different growth factors from the platelets is the mechanism proposed for explaining the regenerative potential of platelet concentrates. Over the past years, platelet-rich plasma (PRP) has been the subject of a lot of study, and its capacity for regeneration is widely recognized. Platelet-rich fibrin (PRF), a second generation platelet concentrate, develops when trapped platelets, leukocytes, growth factors, glycoproteins, and cytokines are encircled by a dense fibrin network. PRF’s physiological architecture encourages stem cell attraction, migration, and differentiation, which aid in wound healing [15–19]. Studies have demonstrated the antimicrobial activity of platelet concentrates against several kinds of bacteria, such as Klebsiella pneumoniae, Escherichia coli, Staphylococcus aureus, and Streptococcus oralis. Leukocytes found in these concentrates are considered to be the source of this antimicrobial effect [20, 21].

The combined application of leukocyte platele rich fibrin (L-PRF) and metronidazole in treatment of periodontitis has not been previously evaluated. Thus, this study aimed to assess the impact of using the L-PRF as an antibiotic local delivery biological device adjunctive to non-surgical periodontal therapy for the management of moderate periodontitis both clinically and micro-biologically. The null hypothesis of the study was that there would be no differences in clinical parameters or microbiological parameters of the periodontium between groups treated by L-PRF alone or L-PRF loaded with Metronidazole.

## Methods

### Ethical approval and informed consent

#### Ethical approval

was granted from the Research Ethics Committee, Faculty of Dentistry, Alexandria University (IRB No. 0413-2/2022, IORG 0008839). The study was carried out in accordance with the principles of the modified Helsinki code for human clinical studies (2013) [22]. Before any interventions, the nature and purpose of the study were explained to the participants, who then provided their written, informed consent.

### Study design

**Fig. 1:**
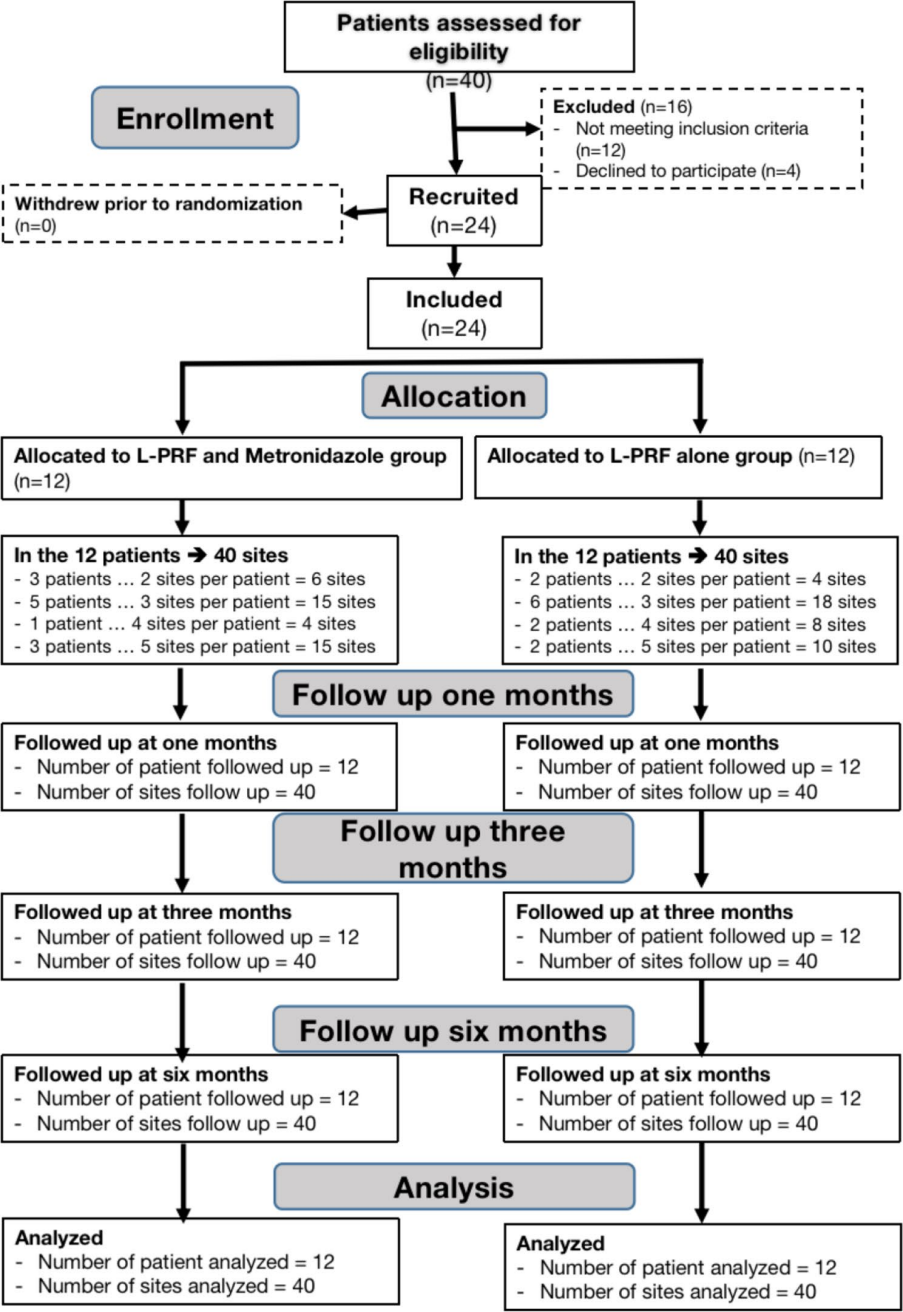
Consort flow chart 2010

This randomized controlled clinical trial has been carried out in accordance with the Consort guidelines (2010) [23] (Fig. 1). From June 2022 to October 2023, the study was conducted at the Department of Oral Medicine, Periodontology, Oral Diagnosis, and Oral Radiology, Faculty of Dentistry, Alexandria University, Egypt.

### Sample size calculation

Based on the assumptions of 5% alpha error and 80% study power, the sample size was estimated. The standard deviation (SD) percent reduction in log CFU/ml in *P. gingivalis* was calculated to be 21.10% (3.67) and 16.45% (0.515) for Metronidazole and PRF, respectively. Based on comparing two dependent means using the highest SD of 3.67%, the minimum sample size was calculated to be 11 patients per group, increased to 12 patients to make up for lost to follow up cases. Total sample = number per group x number of groups = 12 × 2 = 24 patients. G*Power for Windows (version 3.1.9.7) was used to calculate the sample size, which was based on Rosner’s method [24].

### Participants

Twenty-four patients with eighty periodontal pockets had moderate periodontitis and were recruited from the outpatient clinic of the Department of Oral Medicine, Periodontology, Oral Diagnosis, and Oral Radiology. Faculty of Dentistry, Alexandria University.

### Inclusion criteria

I. Moderate periodontitis with attachment loss of 3–4 mm, probing depth ≤ 5, and bleeding on probing [25].
II. Patients age from twenty-five to fifty.
III. No history of periodontal therapy, either surgical or non-surgical, in the previous six months.
IV. Both sexes.
V. Patients with an O’Leary plaque index ≤ 15% [26].

### Exclusion criteria

I. Smokers.
II. Pregnancy and lactating women.
III. Systemic and debilitating diseases.
IV. Use of antibiotics during the last six months.
V. A previous unfavourable response to the study’s products.
VI. Patients with bleeding disorders [27].
VII. Patients taking antiplatelet therapy and anticoagulant therapy [27].
VIII. Patients with severe hepatic impairment or renal disease [28].

### Randomization and allocation concealment

The participants were divided into the test and control groups through a random allocation process. The allocation sequence was created using computer-generated random allocation software, using the permuted block randomization technique, where participants were allocated in blocks of 4 [29].

An independent individual responsible for the trial sealed the allocation sequence in opaque envelopes and only opened them at the time of treatment [30]. It was not possible to blind the participants and the operator due to variations among the two techniques. One operator carried out all the steps, while a different examiner conducted the outcome assessment. The reliability of measurements taken by the same examiner was determined, and it was found that the intraclass correlation coefficient (ICC) was 0.91, which demonstrates very good reliability [31].

### Pre-procedure preparation

All patients underwent phase I therapy to achieve optimum plaque control and improve gingival health. Patients were instructed to implement plaque control measures and re-evaluated two weeks after initial treatment.

### Outcomes assessment

#### Microbiological parameters

Microbiological relative quantitation of P. gingivalis before treatment and after one month of treatment was performed by quantitative real-time PCR (qPCR). The sampling was performed by aseptically inserting sterile paper points into the pockets of the periodontal tissue. Paper points were then put into sterile cryovials and stored immediately at -80 °C until the time of DNA extraction. Total DNA extraction from paper points was done using the QIAamp^®^ DNA Mini Kit (Qiagen) [32]. Maxima SYBR Green qPCR (ThermoScientific, Baltics UAB) was used for relative quantitation of PG DNA by real-time PCR using Universal 16s rDNA bacterial primers and P. gingivalis-specific primers (ThermoFisher Scientific, UAB) [32]. The delta-delta CT method was followed for relative quantitation of P. gingivalis [33].

#### Clinical parameters

Probing depth (PD) [34], bleeding index (BI) [34], plaque index (PI) [35], modified gingival index (MGI) [36], and clinical attachment loss (CAL) [36] were measured in all patients at baseline and at one, three, and six months following phase I therapy. The PD was measured to the nearest whole millimetre from the gingival margin to the most apical gingival tissue penetration of the periodontal probe tip at a 45° angle to the long axis of the tooth surface using William’s calibrated periodontal probe, with the probe inserted with its long axis aligned parallel to the long axis of the tooth. Interproximal PD measurements were carried out immediately adjacent to interproximal tooth contact points. If there was no interproximal contact present, the periodontal sites were excluded from analysis. And The CEJ-GM distance was measured to the nearest whole millimetre, with positive numbers recorded if the most coronal aspect of the gingival tissue margin was located on enamel and negative values recorded when the most coronal aspect of the gingival tissue margin was located apical to the CEJ on cementum. CAL = (PD) - (CEJ-GM) Only periodontal sites where the CEJ could be clinically located were included in the present analysis [37].

### PRF preparation method

#### L-PRF preparation

The procedure for preparing the PRF was carried out according to Choukroun et al. (2001) [38]. Twelve patients had venous punctures to obtain blood samples into 10 ml plain glass tubes free of anticoagulants (PRF Process, Sum-bow blood collection tube, China). The tubes were then immediately centrifuged using a fixed-angle centrifuge (PRF Process, Tangkula 800-1, China) at 2,700 rpm for 12 min at room temperature. The fibrin clot in the middle fraction of the centrifuged blood was extracted with sterile tweezers, and the red blood cell (RBC) base was separated from it using sterile scissors. L-PRF was then formed into a membrane by squeezing out the fluids that were inside the fibrin clot.

#### L-PRF preparation loaded with metronidazole incorporation

PRF was prepared according to the previously described protocol [38]. Twelve patients had venous punctures to obtain blood samples into 10-millilitre plain glass tubes devoid of anticoagulants (PRF Process, Sumbow blood collection tube, China) [38]. Before the tubes were centrifuged, metronidazole (250 mg tablets, Sanofi Aventis, France) was dissolved in 25 ml of saline. A sterile syringe was then used to add 0.5 ml of the previously made metronidazole solution to 10 ml of blood sample [38]. After that, the tubes were centrifuged at room temperature for twelve minutes at 2,700 rpm using a fixed-angle centrifuge (PRF Process, Tangkula 800-1, China). After centrifugation, the fibrin clot found in the middle fraction of the centrifuged blood was collected using sterile tweezers and separated from the RBC base using sterile scissors, following the same procedure for the preparation of the PRF membrane.

### Interventions

#### Group I

**Fig. 2:**
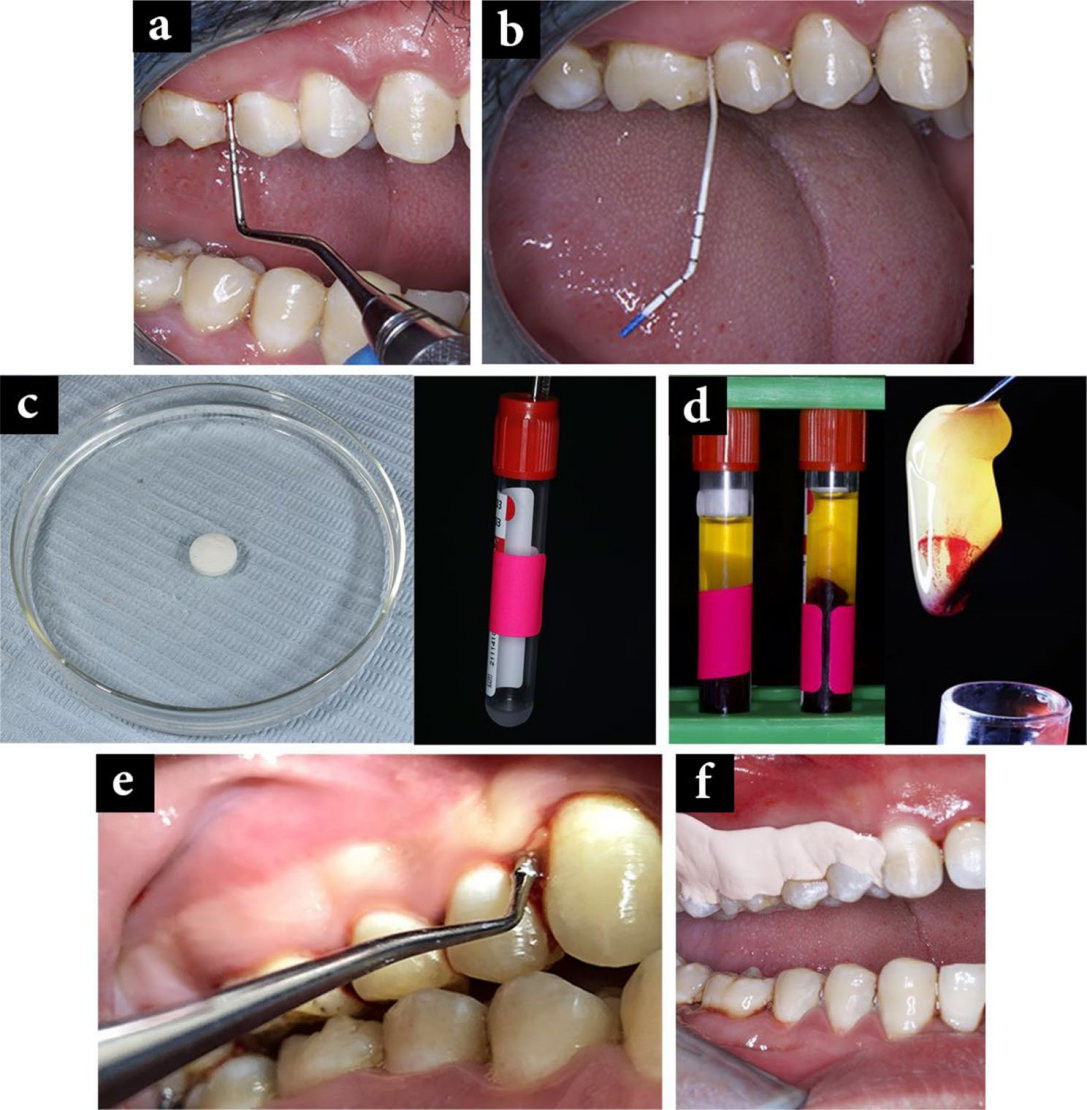
Test group: **a**) Probing depth at baseline, **b**) Microbiological sampling at baseline, **c**) 0.5 ml metronidazole mixed with L-platelet -rich fibrin, **d**) Platelet rich fibrin preparation, **e**) Placement of L-PRF, **f**) Perio -pack application

L-PRF-loaded metronidazole membrane was applied subgingivally into the pockets using a plastic filling instrument (2 Woodson instrument, Hu-Friedy Manufacturing Co LLC, Chicago, IL, USA), also known as placement instruments, which is highly polished stainless steel designed for placing and contouring restorative materials into cavity preparations and other dental procedures. These instruments generally have rounded ends that help in applying restoratives without damaging sensitive tissue [39], and then Perio-pack was applied to cover the material (Fig. 2).

#### Group II

**Fig. 3:**
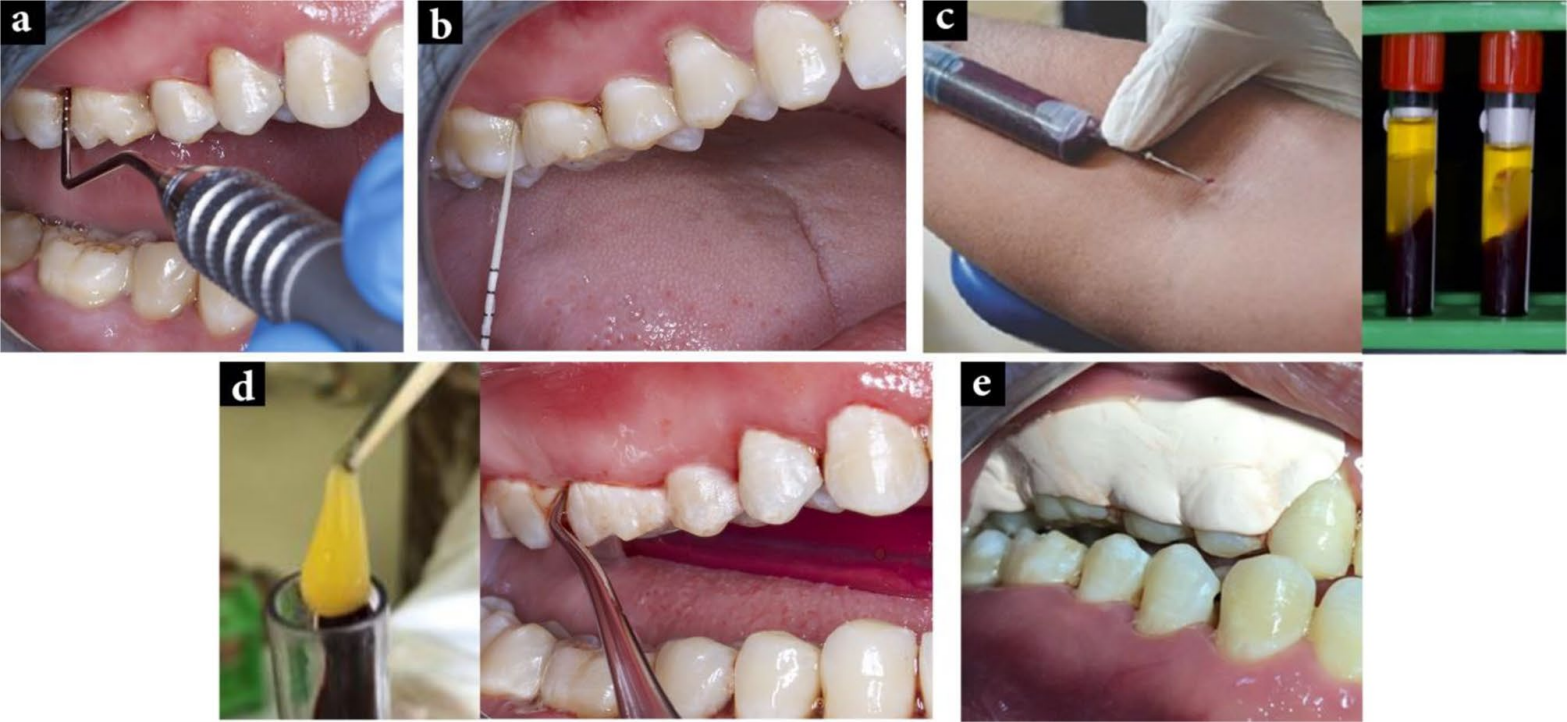
Control group: **a**) Probing depth at baseline, **b**) Microbiological sampling at baseline, **c**) Platelet rich fibrin preparation, **d**) Placement of L-PRF, **e**) Perio -pack application

The L-PRF alone was applied subgingivally into the pockets, and then the Perio-pack was applied to cover the material in the pocket (Fig. 3).

### Post-procedures care

- The patients were given instructions to follow a dental hygiene regimen, which includes brushing their teeth twice a day with modified Stillman technique (Modified Stillman’s brushing technique is indicated in patients with gingivitis, periodontitis, and gingival recession, the method was short and soft zigzag vibration by moving the vibration from apical to coronal direction, and then the rolling movement added) [40, 41]. as well as avoiding the perio-pack site. In addition, they were instructed not to chew anything sticky or hard while the gel was being placed.
- After a week, both processes were carried out again for the second application (a second application was done as recommended by the study of Leiknes et al.) [42]. Generally, the application of L-PRF is considered safe as it is formed by autologous blood, promotes healing, and is easily prepared by chairside [43].

### Statistical analysis

The data was analyzed using the statistical package for the social sciences (SPSS) program, version 25 [44]. Since the data are not normally distributed, they were described using the minimum, maximum, median, and a 95% confidence interval (CI) for the median. Statistical significance was determined with a p-value of less than.05.

## Results

Out of forty patients who were evaluated for eligibility, only twenty-four were included in the current study, as shown by the consort flow chart in (Fig. 1). According to Table 1, there were no appreciable variations between the two groups’ demographic features. The majority of participants were male, with a median age of 39.00 in study group and 43.00 in control group. There are no adverse effects observed when adding L-PFR to the metronidazole solution during the three observation periods.

**Table 1:** Shows the age and gender distribution between the studied groups for patients

	L – PRF and Metronidazole (*n* = 12)	L – PRF alone (*n* = 12)	*p* value
Age (years)			
- Min – Max	26.00–50.00	30.00–47.00	*p* = .862 NS
- Median	39.00	43.00	
- 95% CI of the median	32.00–48.00	34.00–46.00	
- 25th – 75th percentile	32.50–47.00	35.00–45.50	
**Sex**			
- Male	8 (66.67%)	6 (50.00%)	
- Female	4 (33.33%)	6 (50.00%)	*p* = .408 NS

n : Number of patients Min-Max: Minimum – Maximum

CI: Confidence interval MW: Mann-Whitney U test

* : Statistically significant (*p* < .05) NS: Statistically not significant (p *≥* .05)

### Clinical parameters

**Fig. 4:**
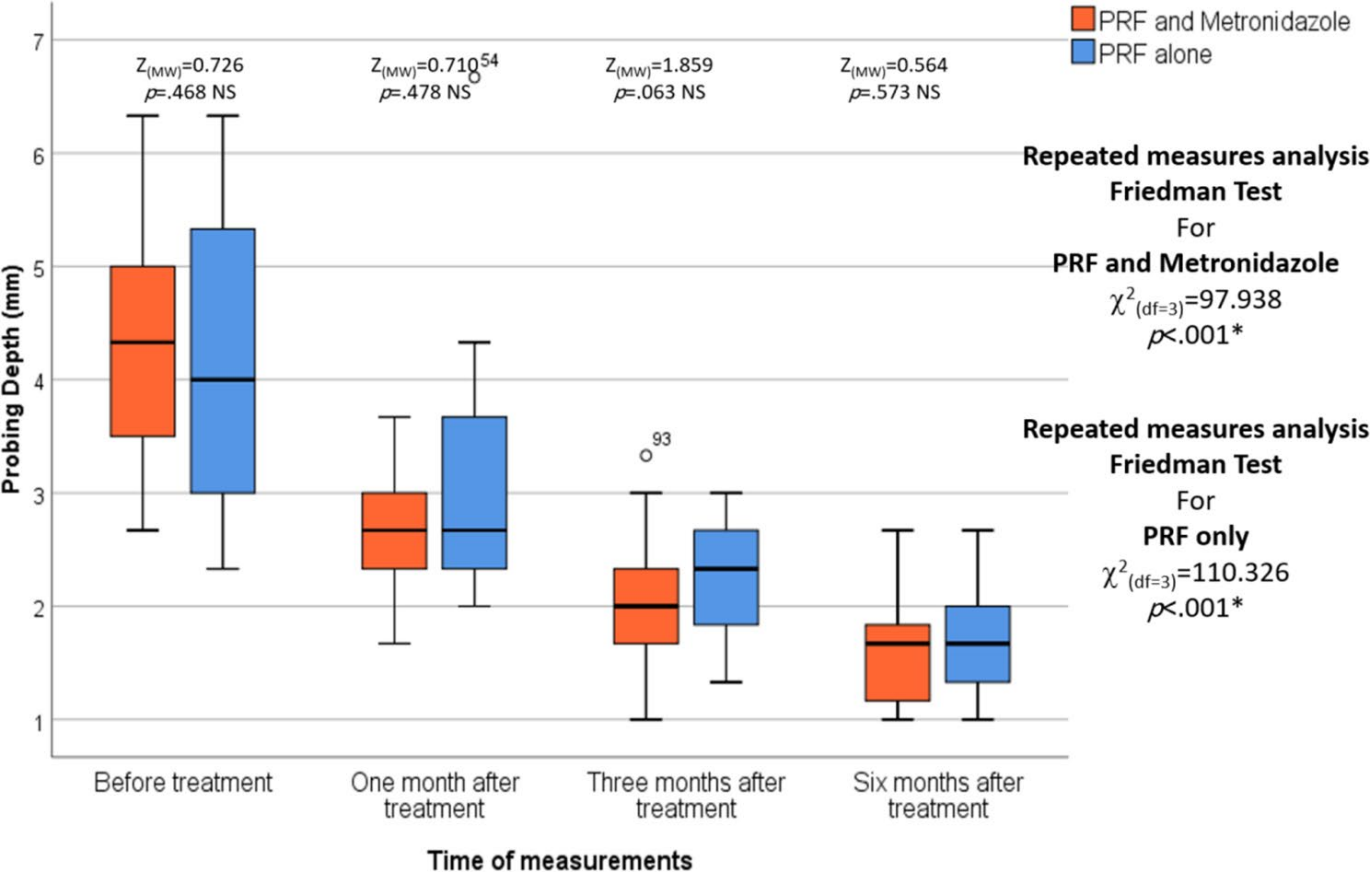
The figure of probing depth shows the differences between the test and control groups over various periods of time .The median is represented by the thick line in the center of the box

**Fig. 5:**
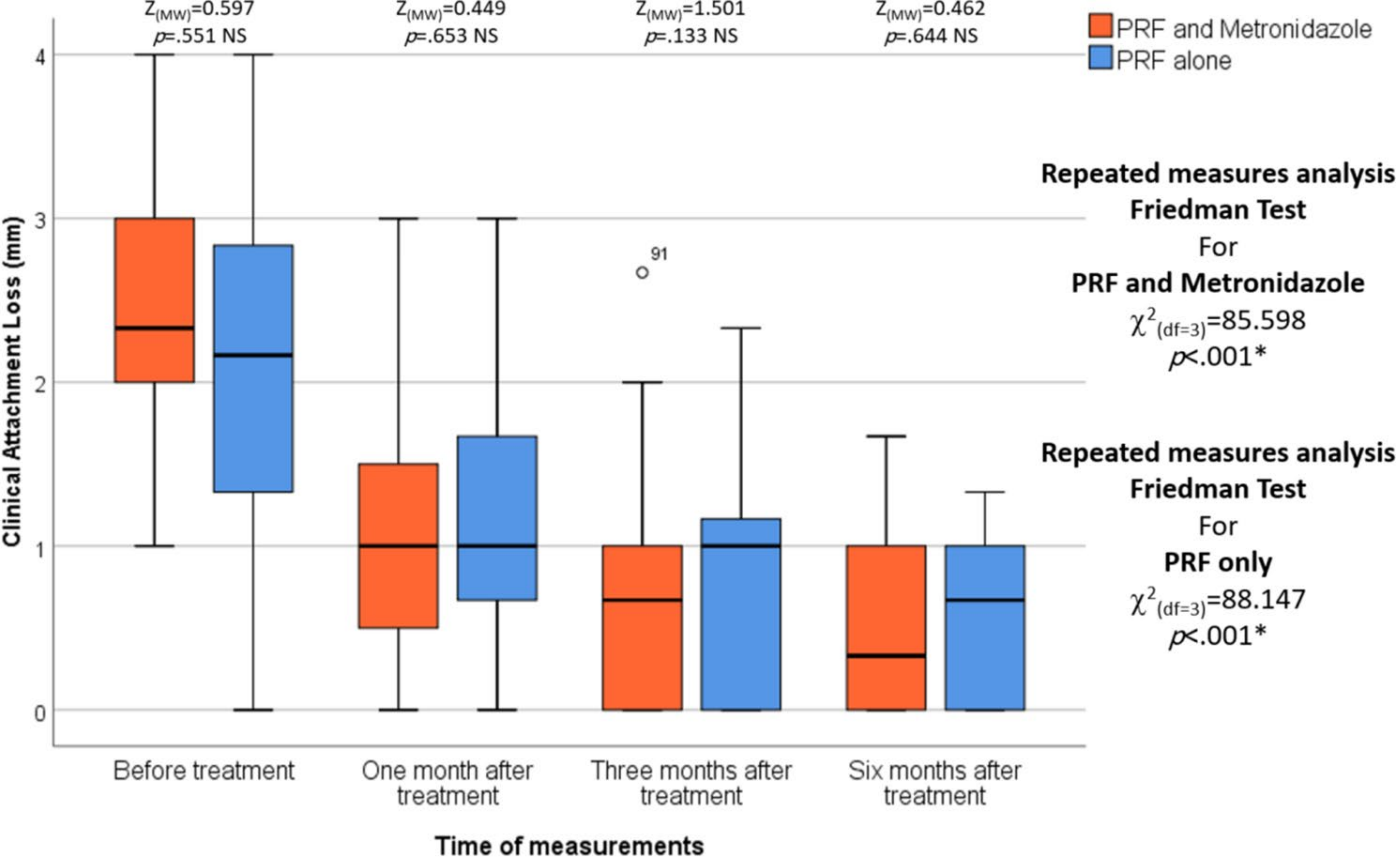
The figure of Clinical attachment loss shows the differences between the test and control groups over various periods of time.The median is represented by the thick line in the center of the box

There were no statistically significant differences between the L-PRF and Metronidazole groups and the L-PRF-only groups in the PD reduction (*p* = .468, *p* = .478, *p* = .063 and *p* = .573,* respectively)* and CAL gain (*p* = .551, *p* = .653, *p* = .133, and *p* = .644, respectively) at all time points. In each group, repeated measures analysis revealed a statistically significant decrease in the CAL and PD among the different measurement times in the L-PRF and Metronidazole group and in the L-PRF-only group (*p* < .001 and *p* < .001, respectively). For both studied groups, pairwise comparisons of different measurement points revealed that PD and CAL after one, three, and six months were statistically significantly lower than before treatment (*p* = .001, *p* < .001, and *p* < .001, respectively). (Table 2), (Figs. 4 and 5)

**Fig. 6:**
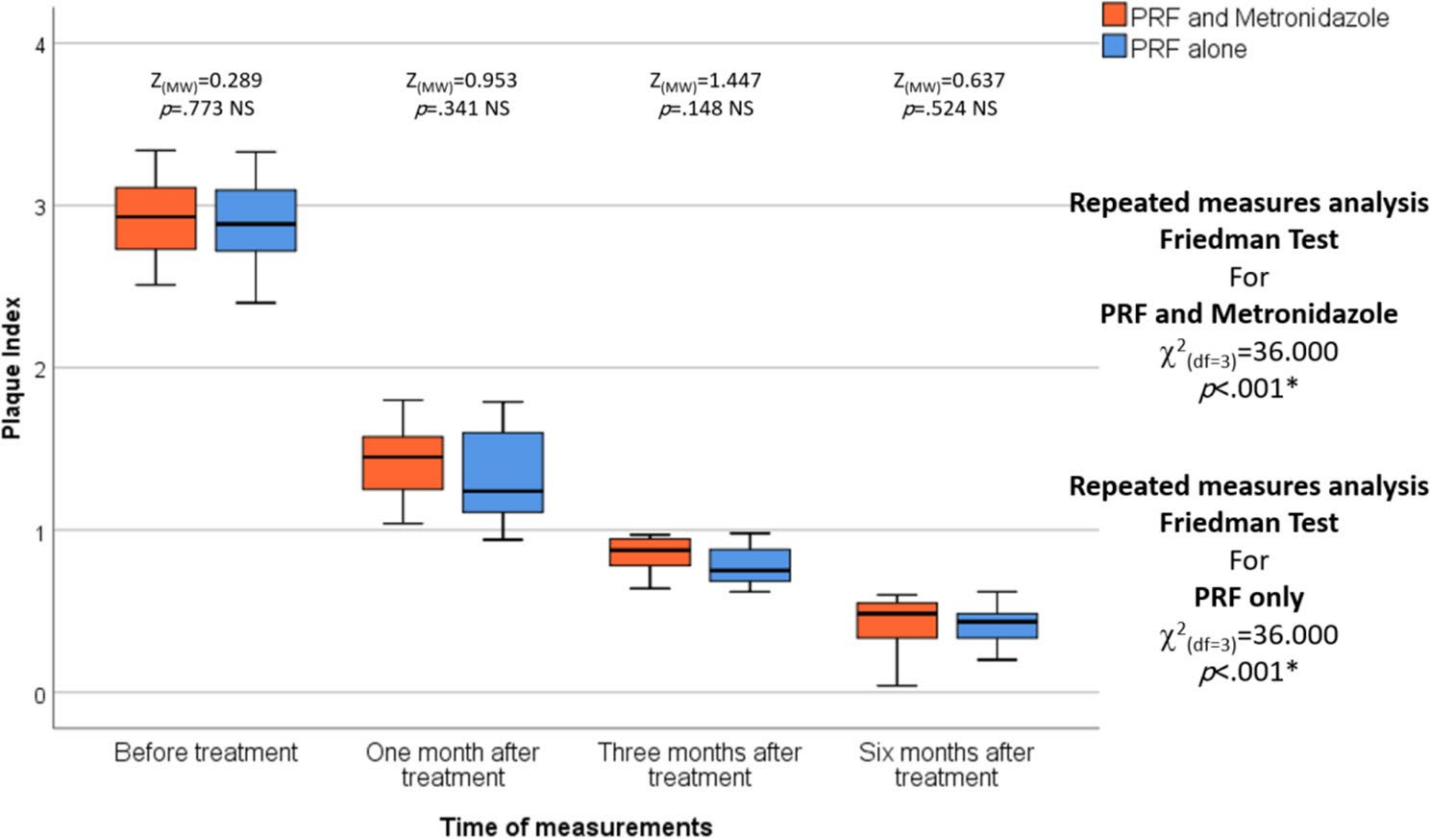
The figure of the Plaque Index shows the differences between the test and control groups over various periods of time, and the median is represented by the thick line in the centre of the box

**Fig. 7:**
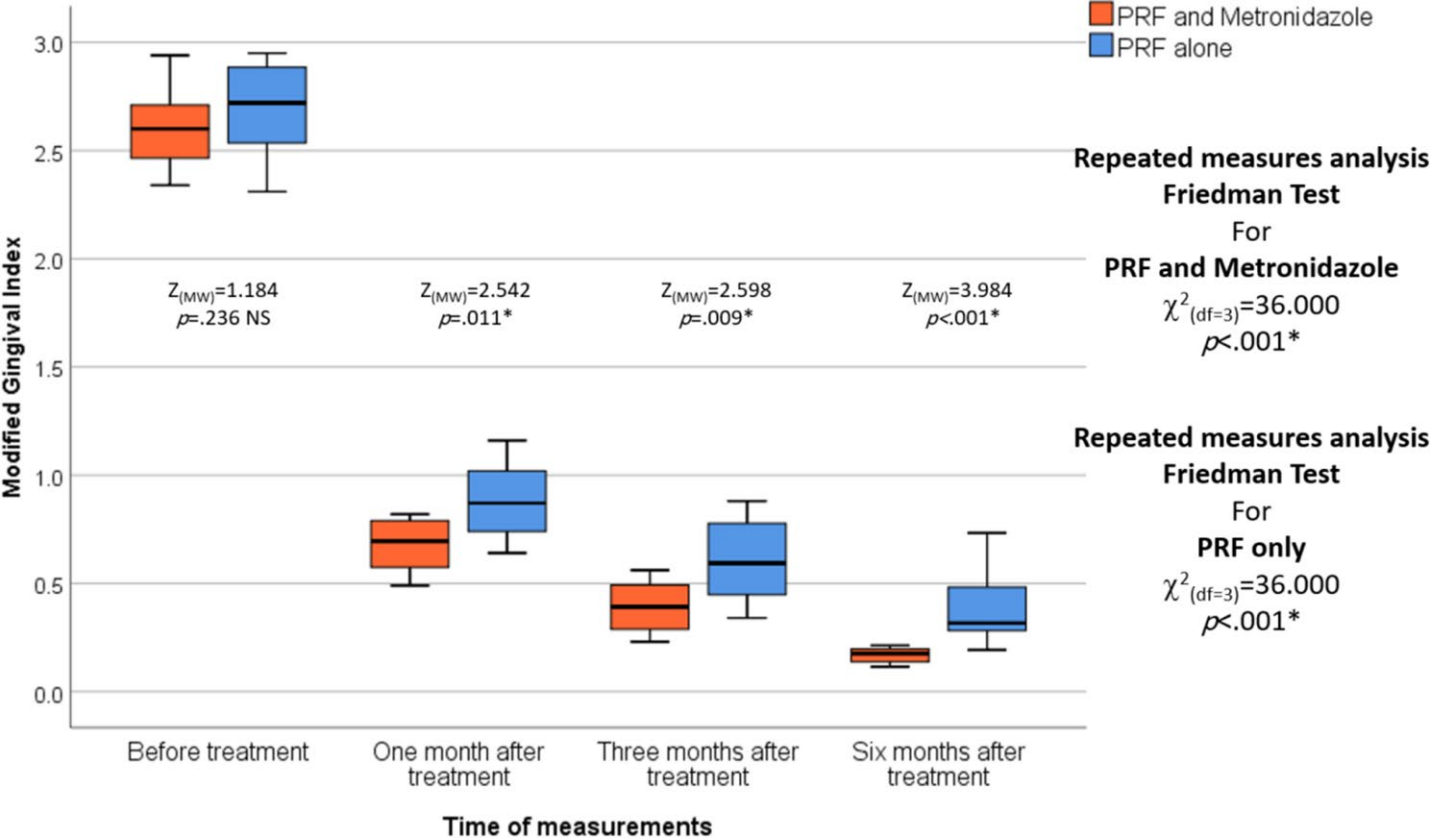
The figure of the modified gingival index shows the differences between the test and control groups over various periods of time, and the median is represented by the thick line in the centre of the box

**Fig. 8:**
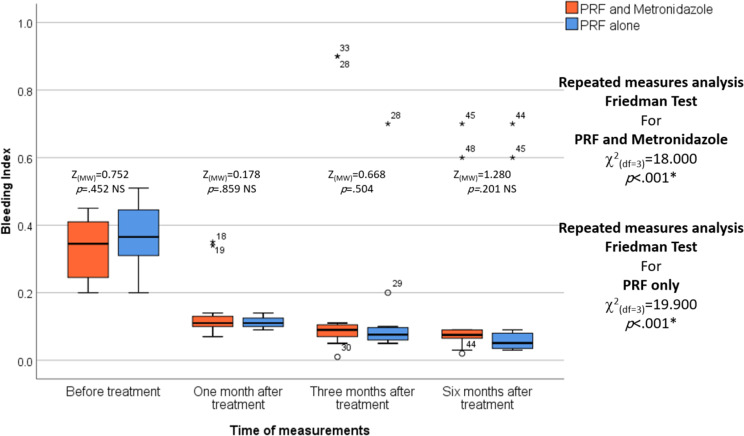
The figure of Bleeding Index index shows the differences between the test and control groups over various periods of time, and the median is represented by the thick line in the centre of the box

No statistically significant differences were observed between the two studied groups in the PI, BI, and MGI before treatment. MGI was statistically significantly lower in the L-PRF and Metronidazole groups compared to the L-PRF-only group after one, three, and six months of treatment (*p* = .011, *p* = .009, and *p* < .001, respectively). In each group, repeated measures analysis revealed a statistically significant decrease in PI, BI, and MGI among the different measurement times in the L-PRF and Metronidazole groups and the L-PRF-only group (*p* < .001 and *p* < .001, respectively). (Table 3), (Figs. 6, 7 and 8).

**Table 2:** A comparison of probing depth and clinical attachment loss between two study groups for different sites

	Group		Test of significance *p* value	
	PRF and Metronidazole (*n* = 40)	PRF alone (*n* = 40)		
Probing Depth mm				
**Before treatment**				
- Min – Max	2.67–6.33	2.33–6.33	Z _(MW)_ = 0.726 *p* = .468 NS	
- Median	4.33^**a**^	4.00^**a**^		
- 95% CI of the median	3.67–5.00	3.33–5.00		
- 25th – 75th percentile	3.50–5.00	3.00–5.33		
**One month after treatment** - Min – Max	1.67–3.67	2.00–6.67	Z _(MW)_ = 0.710 *p* = .478	
- Median	2.67^**b**^	2.67^**b**^		
- 95% CI of the median	2.67–3.00	2.67–3.33		
- 25th – 75th percentile	2.33–3.00	2.33–3.67		
**Three months after treatment**				
- Min – Max	1.00–3.33	1.33–3.00	Z _(MW)_ = 1.859 *p* = .063 NS	
- Median	2.00^**c**^	2.33^**c**^		
- 95% CI of the median	1.67–2.33	2.33–2.67		
- 25th – 75th percentile	1.67–2.33	1.84–2.67		
**Six months after treatment**				
- Min – Max	1.00–2.67	1.00–2.67	Z _(MW)_ = 0.564 *p* = .573 NS	
- Median	1.67^**c**^	1.67^**d**^		
- 95% CI of the median	1.67–2.00	1.67–2.00		
- 25th – 75th percentile	1.17–1.84	1.33–2.00		
**Friedman Test of Significance** ***p*** **value**	χ^2^_(df=3)_ = 97.938 *p < .*001*	χ^2^_(df=3)_ = 110.326 *p < .*001*		
**PD percentage change % (one month vs. before treatment)**				
- Min – Max	-66.67–12.36	-63.19–12.36	Z _(MW)_ = 2.050 *p* = .040*	
- Median	-36.51	− 29.74		
- 95% CI of the median	-43.71 – -27.25 -48.22 − -21.08	-33.40 – -24.95 -37.85 – -20.62		
- 25th – 75th percentile				
**PD percentage change % (three months vs. before treatment)**				
- Min – Max	-80.00 – -12.73	-70.55–0.00	Z _(MW)_ = 2.229 *p* = .026*	
- Median	− 54.50	-45.47		
- 95% CI of the median	-56.29 – -46.60	− 52.61 – -39.94		
- 25th – 75th percentile	-60.03 – -45.26	− 56.73 – -35.39		
**PD percentage change % (Six months vs. before treatment)**				
- Min – Max	-80.00 – -37.45	-76.54 – -33.33	Z _(MW)_ = 1.406 *p* = .160 NS	
- Median	-66.60	-61.27		
- 95% CI of the median	-66.67 – -61.17	-66.60 – -57.08		
- 25th – 75th percentile	-69.32 – -57.05	-66.75 – -54.29		
**Clinical Attachment Loss mm**				
**Before treatment**				
- Min – Max	1.00–4.00	0.00–4.00	Z _(MW)_ = 0.597 *p* = .551 NS	
- Median	2.33^**a**^	2.17^**a**^		
- 95% CI of the median	2.33–3.00	1.67–2.67		
- 25th – 75th percentile	2.00–3.00	1.33–2.84		
**One month after treatment**				
- Min – Max	0.00–3.00	0.00–3.00	Z _(MW)_ = 0.449 *p* = .653 NS	
- Median	1.00^**b**^	1.00^**b**^		
- 95% CI of the median	1.00–1.33	1.00–1.67		
- 25th – 75th percentile	0.50–1.50	0.67–1.67		
**Three months after treatment**				
- Min – Max	0.00–2.67	0.00–2.33	Z _(MW)_ = 1.501 *p* = .133 NS	
- Median	0.67^**c**^	1.00^**c**^		
- 95% CI of the median	0.00–1.00	1.00–1.67		
- 25th – 75th percentile	0.00–1.00	0.00–1.67		
**Six months after treatment**				
- Min – Max	0.00–1.67	0.00–1.33	Z _(MW)_ = 0.462 *p* = .644 NS	
- Median	0.33 ^**c**^	0.67^**c**^		
- 95% CI of the median	0.00–0.67	0.33–1.00		
- 25th – 75th percentile	0.00–1.00	0.00–1.00		
**Friedman Test of Significance** ***p*** **value**	χ^2^_(df=3)_ = 85.598 *p < .*001*	χ^2^_(df=3)_ = 88.147 *p < .*001*		
**CAL percentage change % (one month vs. before treatment)**			Z _(MW)_ = 1.307 *p* = .191 NS	
- Min – Max	-100.00–33.00	-100.00–0.00		
- Median	-50.09	-50.00		
- 95% CI of the median	-63.76 – -42.92	-50.75 – -33.25		
- 25th – 75th percentile	-71.24 − 31.88	-59.88 − -25.00		
**CAL percentage change % (three months vs. before treatment)**			Z _(MW)_ = 1.447 *p* = .148 NS	
- Min – Max	-100.00–0.00	-100.00–0.00		
- Median	-70.95	-62.55		
- 95% CI of the median	-100.00 – -62.55	-67.00 – -50.00		
- 25th – 75th percentile	-100.00 – -57.08	-100.00 – -50.00		
**CAL percentage change % (Six months vs. before treatment)**			Z _(MW)_ = 0.494 *p* = .621 NS	
- Min – Max	-100.00–0.00	-100.00–0.00		
- Median	-87.96	-75.19		
- 95% CI of the median	-100.00 – -66.75	-100.00 – -66.75		
- 25th – 75th percentile	-100.00 – -61.79	-100.00 – -62.55		

n : Number of sites Min-Max: Minimum – Maximum

CI: Confidence interval MW: Mann-Whitney U test

* : Statistically significant (*p* < .05) NS: Statistically not significant (p *≥* .05)

Post-hoc pairwise comparison among time of measurements using Dunn-Sidak method

Different letters denote statistically significant differences between groups using Bonferroni adjusted significance level

**Table 3:** Comparison of the plaque index, modified gingival index, and bleeding index between the two studied groups for patients

	Group		Test of significance *p* value
	PRF and Metronidazole (*n* = 12)	PRF alone (*n* = 12)	
Plaque Index			
**Before treatment**			
- Min – Max	2.51–3.34	2.40–3.33	Z _(MW)_ = 0.289 *p* = .773 NS
- Median	2.93^**a**^	2.89^**a**^	
- 95% CI of the median	2.73–3.12	2.70–3.15	
- 25th – 75th percentile	2.73–3.11	2.72–3.09	
**One month after treatment**			
- Min – Max	1.04–1.80	0.94–1.79	Z _(MW)_ = 0.953 *p* = .314 NS
- Median	1.45^**b**^	1.24^**b**^	
- 95% CI of the median	1.25–1.60	1.10–1.65	
- 25th – 75th percentile	1.25–1.58	1.11– 1.65	
**Three months after treatment**			
- Min – Max	0.64–0.97	0.62–0.98	Z _(MW)_ = 1.447 *p* = .148 NS
- Median	0.88^**b, c**^	0.75^**b, c**^	
- 95% CI of the median	0.74–0.95	0.67–0.92	
- 25th – 75th percentile	0.78–0.95	0.69–0.92	
**Six months after treatment**			
- Min – Max	0.04–0.60	0.20–0.62	Z _(MW)_ = 0.637 *p* = .524 NS
- Median	0.49^**c**^	0.44^**c**^	
- 95% CI of the median	0.31–0.57	0.32–0.51	
- 25th – 75th percentile	0.33–0.55	0.33–0.49	
**Friedman Test of Significance** ***p*** **value**	χ^2^_(df=3)_ = 30.00 *p < .*001*	χ^2^_(df=3)_ = 30.00 *p < .*001*	
**Modified Gingival Index**			
**Before treatment**			
- Min – Max	2.34–2.94	2.31–2.95	Z _(MW)_ = 1.184 *p* = .236 NS
- Median	2.60^**a**^	2.72^**a**^	
- 95% CI of the median	2.53–2.73	2.49–2.90	
- 25th – 75th percentile	2.47–2.71	2.54–2.89	
**One month after treatment**			Z _(MW)_ = 2.542 *p* = .011*
- Min – Max	0.49–0.82	0.64–1.16	
- Median	0.70^**b**^	0.87^**c**^	
- 95% CI for mean	0.57–0.80	0.73–1.03	
- 25th – 75th percentile	0.58–0.79	0.74–1.02	
**Three months after treatment**			
- Min – Max	0.23–0.56	0.34–0.88	Z _(MW)_ = 2.598 *p* = .009*
- Median	0.39^**b, c**^	0.59^**b, c**^	
- 95% CI of the median	0.28–0.50	0.45–0.80	
- 25th – 75th percentile	0.29–0.49	0.45–0.78	
**Six months after treatment**			
- Min – Max	0.12–0.21	0.19–0.73	Z _(MW)_ = 3.984 *p <* .001*
- Median	0.18^**b, c**^	0.32^**b, c**^	
- 95% CI of the median	0.14–0.20	0.28–0.52	
- 25th – 75th percentile	0.14–0.20	0.28–0.48	
**Friedman Test of Significance** ***p*** **value**	χ^2^_(df=3)_ = 36.00 *p < .*001*	χ^2^_(df=3)_ = 36.000 *p < .*001*	
**Bleeding Index**			
**Before treatment**			
- Min – Max	0.20–0.45	0.20–0.51	Z _(MW)_ = 0.752 *p* = .452 NS
- Median	0.35^**a, b**^	0.37^**a, b**^	
- 95% CI of the median	0.24–0.42	0.30–0.45	
- 25th – 75th percentile	0.25–0.41	0.31–0.45	
**One month after treatment**			
- Min – Max	0.07–0.35	0.09–0.14	Z _(MW)_ = 0.178 *p* = .859 NS
- Median	0.11^**a, b**^	0.11^**a, b**^	
- 95% CI of the median	0.10–0.14	0.10–0.13	
- 25th – 75th percentile	0.10–0.13	0.10–0.13	
**Three months after treatment** - Min – Max	0.01–0.90	0.05–0.70	Z _(MW)_ = 0.668 *p* = .504 NS
- Median	0.09^**c**^	0.08^**c**^	
- 95% CI of the median	0.06–0.11	0.06–0.10	
- 25th – 75th percentile	0.07–0.11	0.06–0.10	
**Six months after treatment**			Z _(MW)_ = 1.280 *p* = .201 NS
- Min – Max	0.02–0.70	0.03–0.70	
- Median	0.08^**c**^	0.05^**c**^	
- 95% CI of the median	0.06–0.09	0.03–0.09	
- 25th – 75th percentile	0.07–0.09	0.04–0.08	
**Friedman Test of Significance** ***p*** **value**	χ^2^_(df=3)_ = 18.000 *p < .*001*	χ^2^_(df=3)_ = 19.900 *p < .*001*	

n : Number of patients Min-Max: Minimum – Maximum

CI: Confidence interval MW: Mann-Whitney U test

* : Statistically significant (*p* < .05) NS: Statistically not significant (p *≥* .05)

Post-hoc pairwise comparison among time of measurements using Dunn-Sidak method

Different letters denote statistically significant differences between groups using Bonferroni adjusted significance level

### Microbiological parameter

**Fig. 9:**
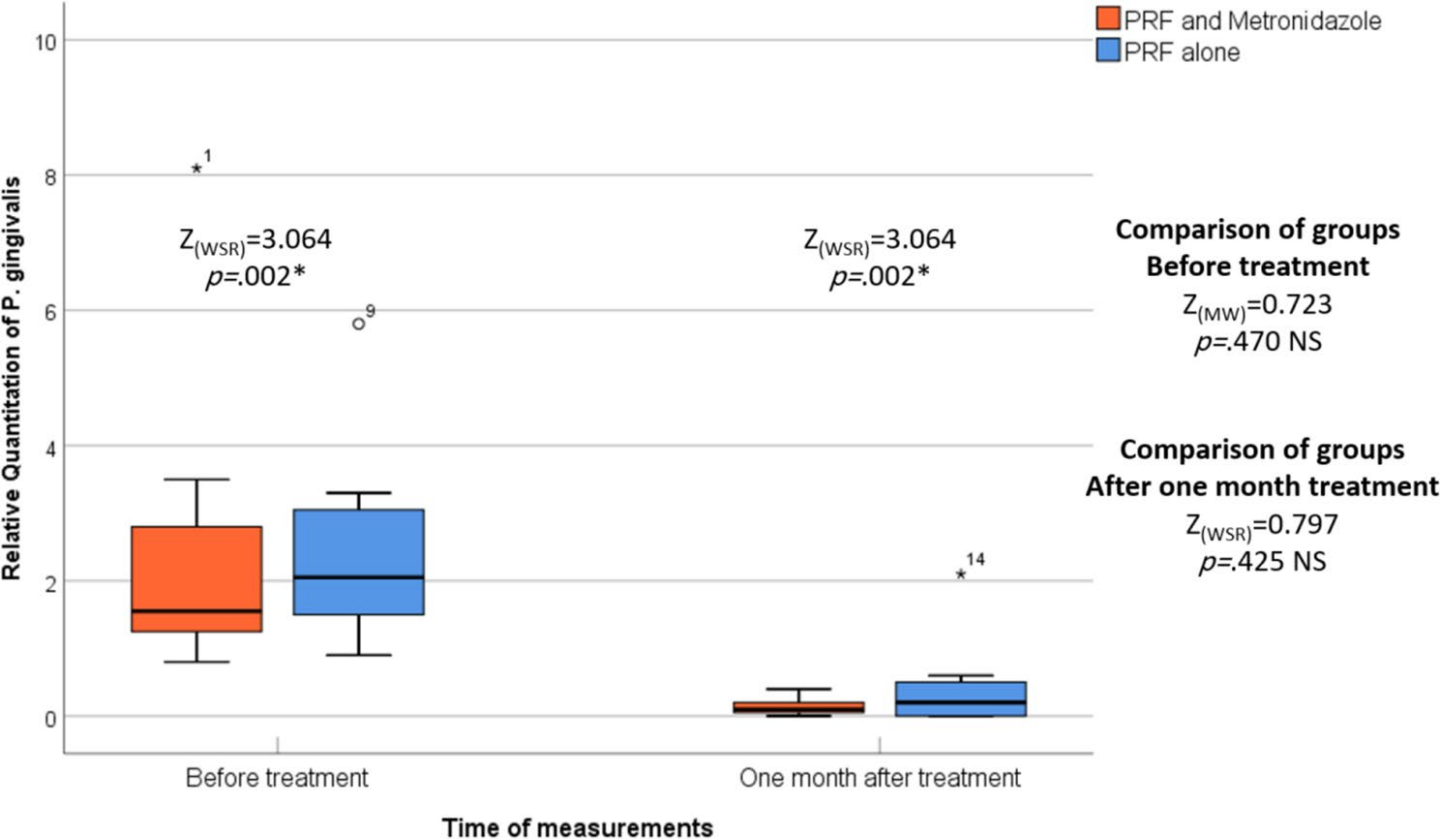
The figure of relative quantitation of Porhyromonas gingivalis shows the differences between the test and control groups over various periods of time, and the median is represented by the thick line in the centre of the box

In both studied groups, the relative quantitation of *P. gingivalis* showed a statistically significant decrease after one month compared to baseline (*p* = .002 and *p* = .002, respectively). Notably, the L-PRF-metronidazole group demonstrated a superior reduction in the relative count of *P. gingivalis* compared to the L-PRF-only group. However, there was no statistically significant difference between the two groups under study. (Table 4), (Fig. 9).

**Table 4:** Comparison of relative quantitation of P. gingivalis between the two studied groups for different sites

Relative Quantitation of *P*. gingivalis	Group		Test of significance *p* value
	PRF and Metronidazole (*n* = 40)	PRF alone (*n* = 40)	
Before treatment			
- Min – Max	0.80–8.10	0.90–5.80	Z _(MW)_ = 0.723 *p* = .470 NS
- Median	1.55	2.05	
- 95% CI of the median	1.10–3.00	1.50–3.20	
- 25th – 75th percentile	1.25–2.80	1.50–3.05	
**One month after treatment**			
- Min – Max	0.00–0.40	0.00–2.10	Z _(MW)_ = 0.797 *p* = .425 NS
- Median	0.10	0.20	
- 95% CI of the median	0.10–0.20	0.00–0.60	
- 25th – 75th percentile	0.05–0.20	0.00–0.50	
**Test of significance** ***p*** **value**	Z _(WSR)_ = 3.064 *p* = .002*	Z _(WSR)_ = 3.064 *p* = .002*	
**Percentage change %**			
- Min – Max	− 100.00 – − 62.50	− 100.00 – − 33.33	Z _(MW)_ = 0.672 *p* = .501 NS
- Median	− 94.74	– 90.47	
- 95% CI of the median	− 100.00 – – 87.50	− 100.00 – − 62.50	
- 25th – 75th percentile	− 99.38 – − 88.04	− 100.00 – − 67.92	

n : Number of sites Min-Max: Minimum – Maximum

CI: Confidence interval MW: Mann-Whitney U test

* : Statistically significant (*p* < .05) NS: Statistically not significant (p *≥* .05)

## Discussion

Periodontitis is the most prevalent oral disease that damages the bones and soft tissues continuously and irreversibly by triggering the immune system [45]. The field of regeneration has recently become more interested in endogenous regenerative technology [14]. These concentrates have demonstrated antibacterial activity against multiple bacteria in addition to their ability to promote wound healing [46, 47].

To the best of our knowledge, no studies have been conducted to evaluate L-PRF as an antibiotic slow-release biological device in the non-surgical treatment of moderate periodontitis. The aim of this study was to evaluate the synergistic effects of this combination at the clinical and microbiological levels as an adjunctive non-surgical therapy for the treatment of moderate periodontitis.

The study findings revealed that treatment with both L-PRF alone and in combination with metronidazole resulted in improvements in clinical parameters such as PD reduction, CAL gain, PI reduction, BI decrease, and MGI reduction when compared to baseline. It was noted that the L-PRF-metronidazole group showed superior results regarding the studied parameters during the three observation periods; however, this improvement was not statistically significant.

Our findings agreed with those of Kadam et al. [48], who investigated the clinical efficacy of L-PRF administered alone and in admixture with amoxicillin in managing intra-bony defects and recorded the clinical parameters 6 months postoperatively. They observed superior results regarding CAL gain and PD reduction. This might have been the outcome of actual periodontal regeneration through new attachment in the case of L-PRF, which agreed with current results. Additionally, our results regarding clinical parameters (PD, CAL) were in agreement with those of Narendran et al. [49], who determined that L-PRF was superior in treating periodontal pockets than SRD alone.

Moreover, consistent with our results, Taneja et al. [12] assessed the effectiveness of L-PRF with MTZ and L-PRF alone in the treatment of intra-bony periodontal defects. Their results indicated that L-PRF with MTZ showed improvements in PI, PD, MGI, and CAL outcomes when compared to L-PRF alone. Growth factors found in platelets are essential for directing regenerative cells to the site of healing.

The current study’s microbiological findings showed that, in comparison to baseline, both treatments reduced the relative quantitation of Porhyromonas gingivalis (PG). After a month of treatment, the L-PRF-metronidazole group notably had better results, but this difference wasn’t of statistical significance.

Previous studies evaluated the application of L-PRF as a vehicle for antimicrobials in various applications in dentistry, although these studies were not identical to our work [50–52]. For instance, the evaluation of PRF as a vehicle for antimicrobials therapy was evaluated by Rafiee et al. [50], who found rapid release of the antimicrobials from the PRF vehicle during the first twenty-four hours, followed by a sustained release for up to 14 days, which agreed with the current results.

Moreover, the results of this study coincide with the findings of Ercan et al. [51], who evaluated the efficacy of PRF as a high-capacity delivery system for antimicrobials and found that PRF has a higher antimicrobial loading capacity and long-acting antibacterial effects.

Our findings agreed with those of Bennardo et al. [53], who evaluated the role of L-PRF as a natural carrier for gentamicin, linezolid, and vancomycin delivery through the analysis of drug release and antimicrobial activity. They reported that vancomycin interfered with L-PRF formation. Vancomycin caused unlimited RBC accumulation at concentrations of 3 mg per mL or higher, but this effect was avoided by the use of sodium citrate. This explains why vancomycin inhibits PRF formation but not PRP formation. The physical characteristics of L-PRF remained unchanged with gentamicin and linezolid.

These findings are consistent with an in vitro study conducted by Polak et al. [54], who assessed the viability of utilizing L-PRF as a locally sustained, released device for antimicrobials. When compared to L-PRF incorporated with saline, they found that L-PRF incorporated with antibiotics before centrifugation demonstrated notable antibacterial activity [55].

On agar plates, the antimicrobial efficacy of L-PRF against periodontal pathogens was assessed by Castro et al.‘s study [56]. According to their report, the L-PRF membrane exhibited strong antimicrobial capabilities against PG.

Furthermore, Kour et al. [57] evaluated the anti-microbial efficacy of PRF, I-PRF, and PRP against PG. Their findings demonstrated that I-PRF had the largest zone of inhibition and was noticeably better than PRF. Moreover, PRP demonstrated a significantly larger zone of inhibition against PRF. This may be the result of PRF having a lower concentration of white blood cells and platelets than other platelet concentrates.

In a study conducted by Siawasch et al. [58] to evaluate the effect of local administration of antimicrobials on leukocyte- and platelet-rich fibrin (L-PRF), They observed no statistically significant difference in the release of PDGF-AB, VEGF, TGF-β1, and BMP-2 at each time point evaluated up to 14 days between L-PRF membranes with and without the incorporation of metronidazole solution.

It is recommended to conduct additional research to evaluate MTZ’s antimicrobial effectiveness in L-PRF at various concentrations. It would also be beneficial to assess the effectiveness of liquid PRF versus solid forms in the treatment of periodontitis. A more complete evaluation would result from extending the microbiological evaluations to the third and sixth months after the start of treatment. Further studies should be carried out to assess the controlled release of metronidazole in the L-PRF metronidazole formula.

## Conclusion

Both treatment modalities, L-PRF with incorporated MTZ and L-PRF alone, demonstrated clinical and microbiological effectiveness in treating moderate periodontitis. However, the combination of MTZ with L-PRF was superior to L-PRF alone in managing periodontal diseases.

## Electronic supplementary material

Below is the link to the electronic supplementary material.

Supplementary Material 1

Supplementary Material 2

Supplementary Material 3

Supplementary Material 4

Supplementary Material 5

Supplementary Material 6

Supplementary Material 7

Supplementary Material 8

Supplementary Material 9

Supplementary Material 10

Supplementary Material 11

Supplementary Material 12

Supplementary Material 13

Supplementary Material 14

Supplementary Material 15

Supplementary Material 16

## Data Availability

No datasets were generated or analysed during the current study.

## Abbreviations

PCR: Polymerase chain reaction
SRD: Scaling and root debridement
RBC: Red blood cell
LDD: Local drug delivery
TET: Tetracycline
DOX: Doxycycline
MIN: Minocycline
MTZ: Metronidazole
CHX: Chlorhexidine
AZM: Azithromycin
CLI: Clindamycin
ERT: Endogenous regenerative technology
PRF: Platelet-rich fibrin
PRP: Platelet-rich plasma
L-PRF: Leukocyte platelet-rich fibrin
PD: Probing depth
CAL: Clinical attachment loss
PI: Plaque index
MGI: Modified gingival index
BI: Bleeding index
PG: Porhyromonas gingivalis
CI: Confidence interval
I-PRF: Injectable platelet–rich fibrin
T-PRF: Titanium platelet–rich fibrin
