# Supplementary figures and images for: Evaluation of leukocyte-platelet rich fibrin as an antibiotic slow-release biological device in the treatment of moderate periodontitis: a randomized controlled clinical trial
Source: BMC Oral Health. 2024 Dec 21;24:1530. doi: 10.1186/s12903-024-05254-x (PMC11662799; doi:10.1186/s12903-024-05254-x)

**Figure (2)**


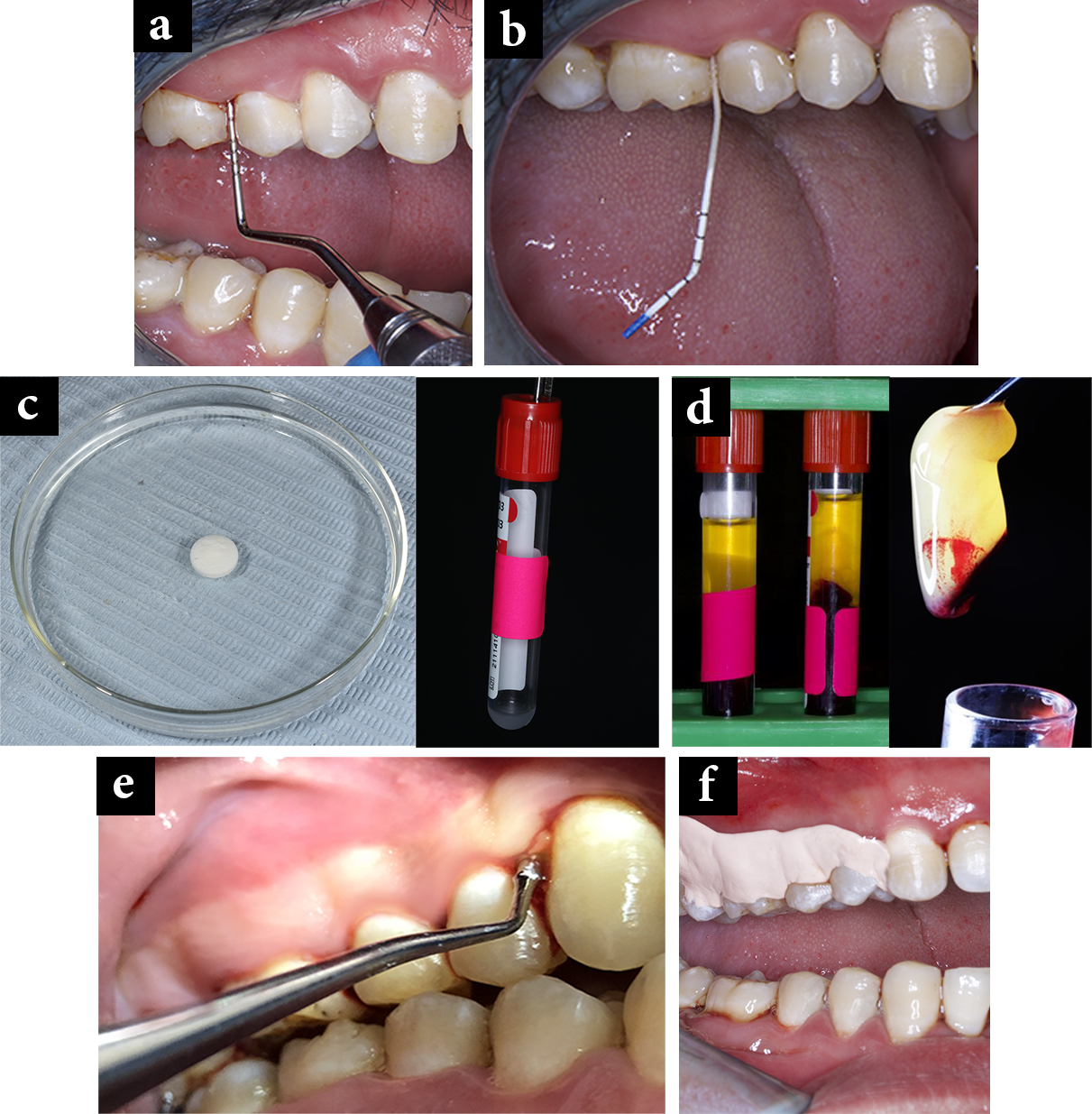


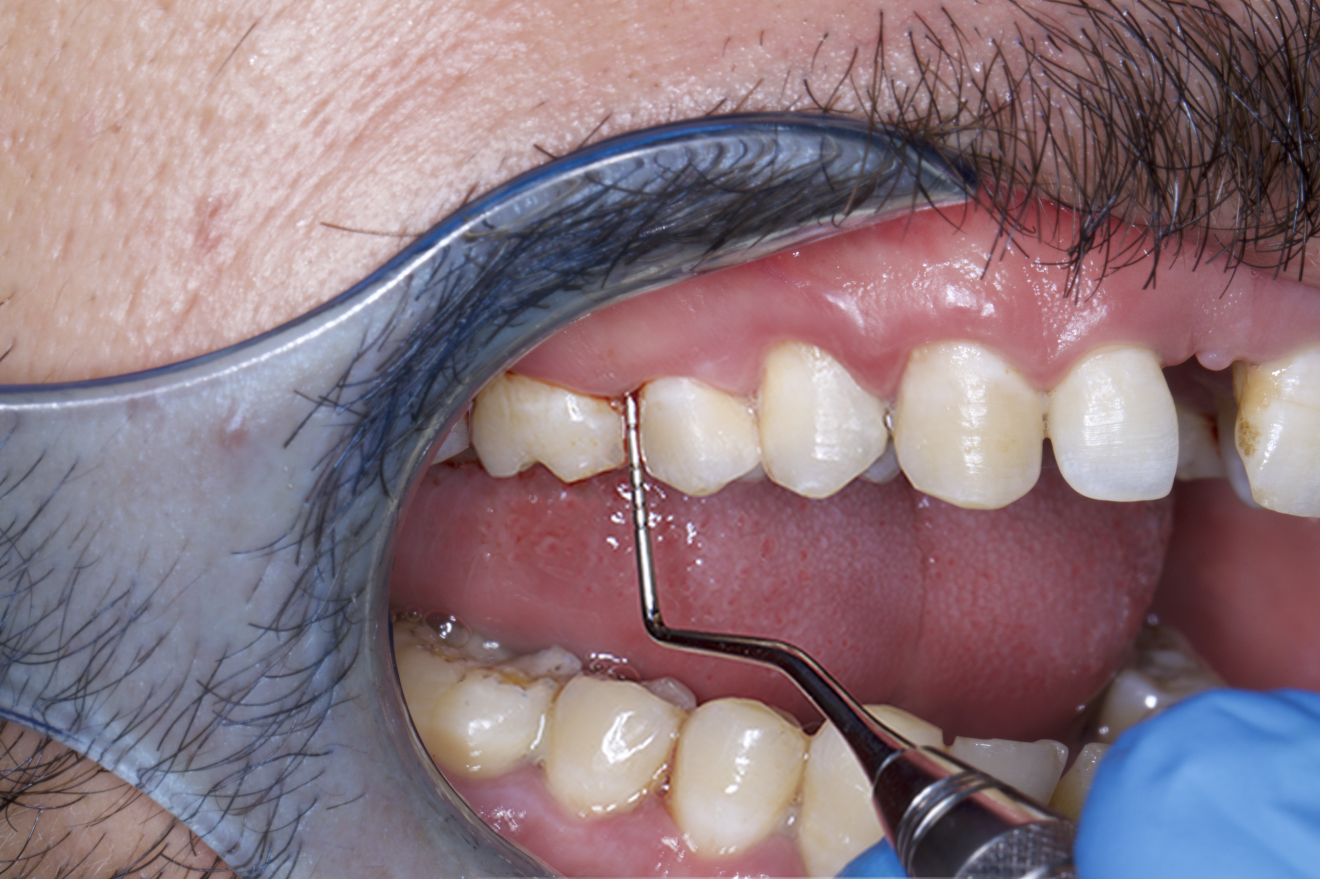


(a)


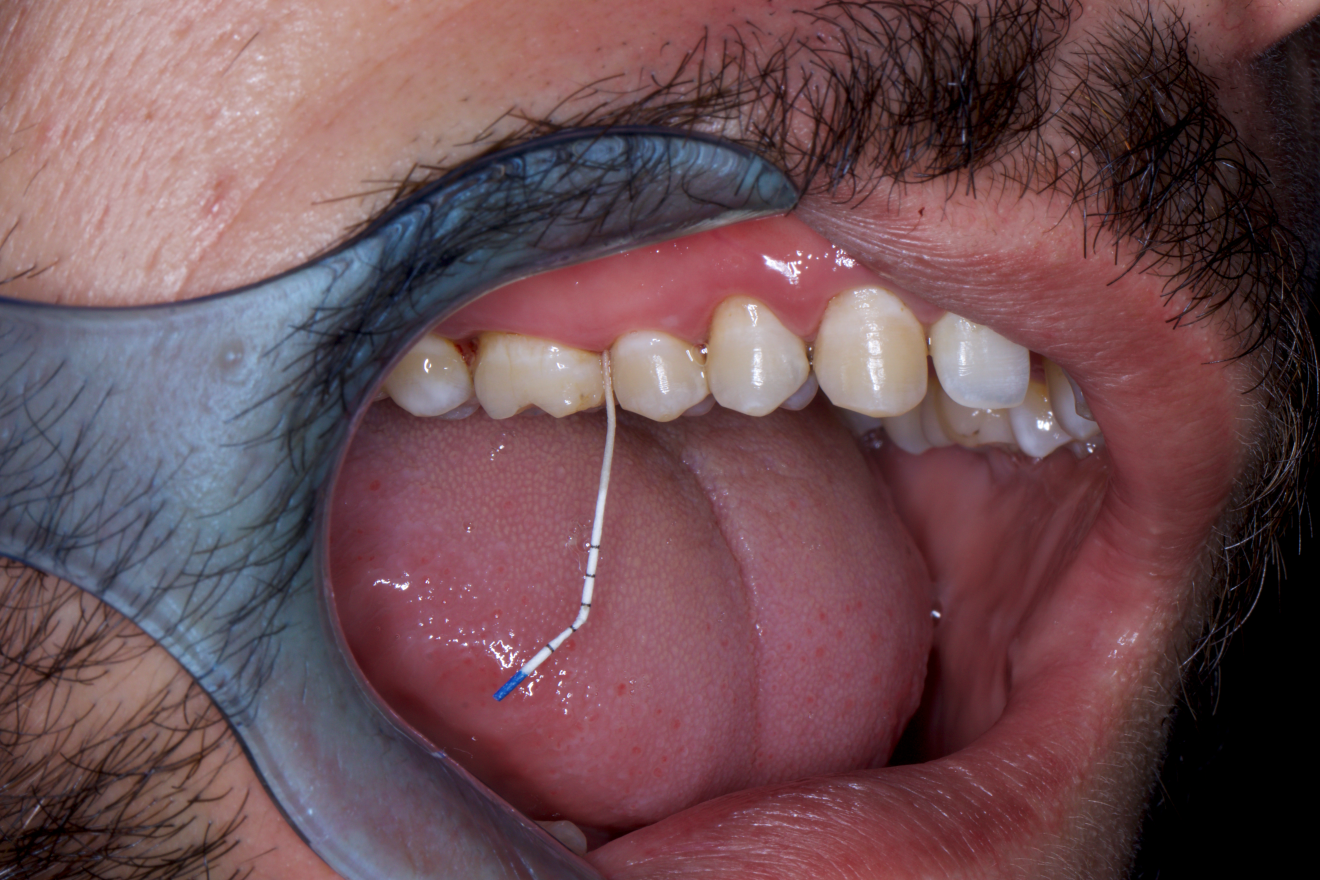


(b)


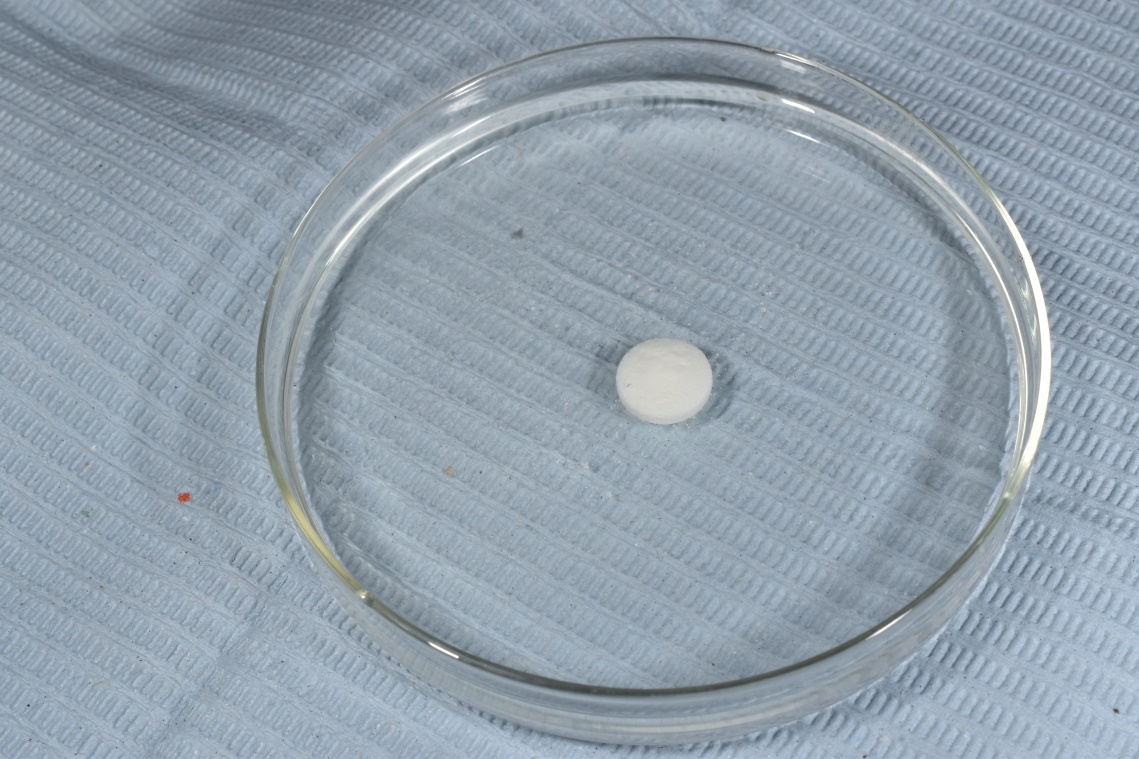

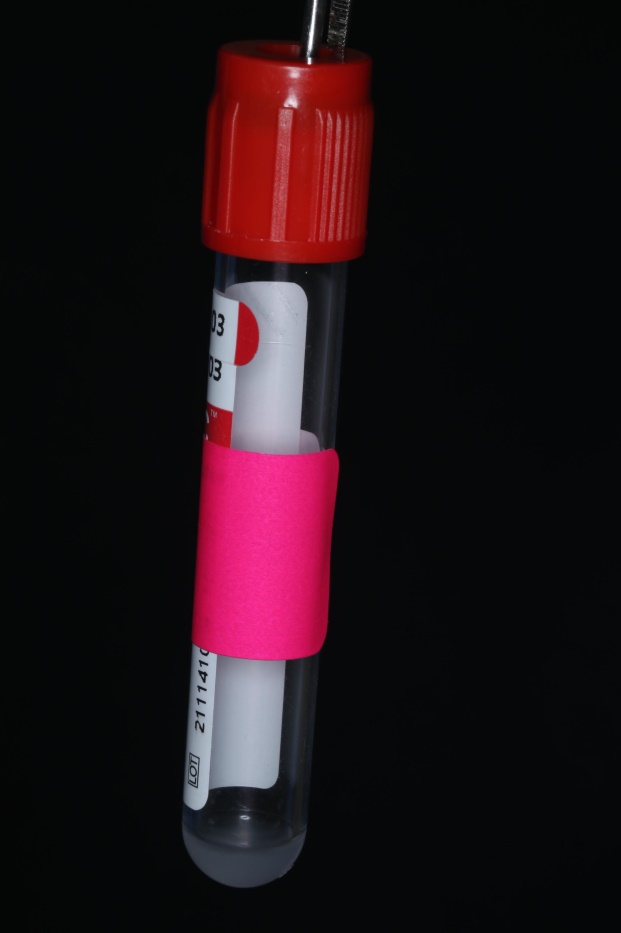


(c)


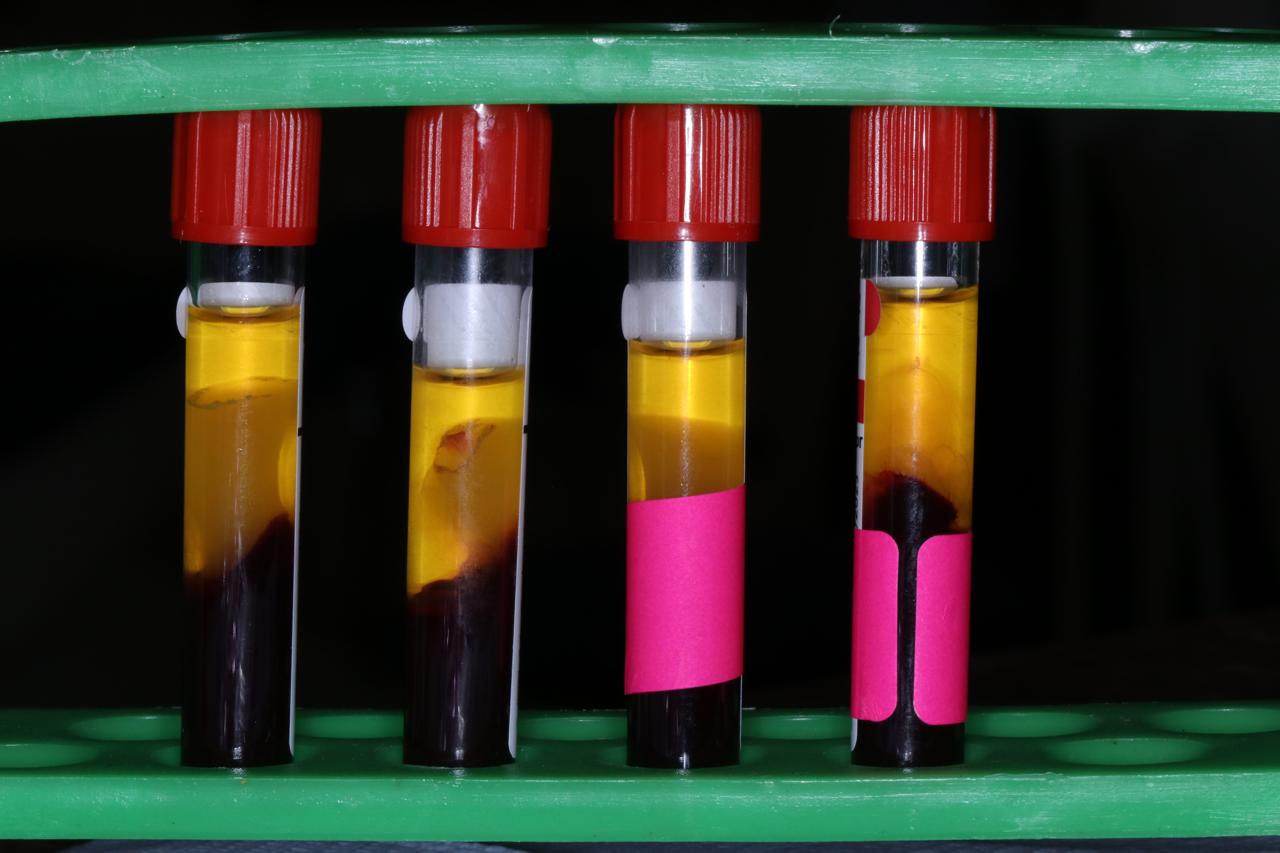

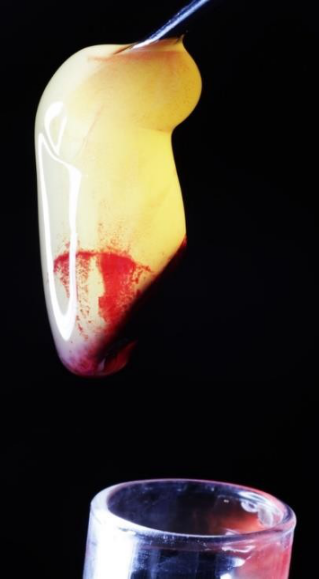


(d)


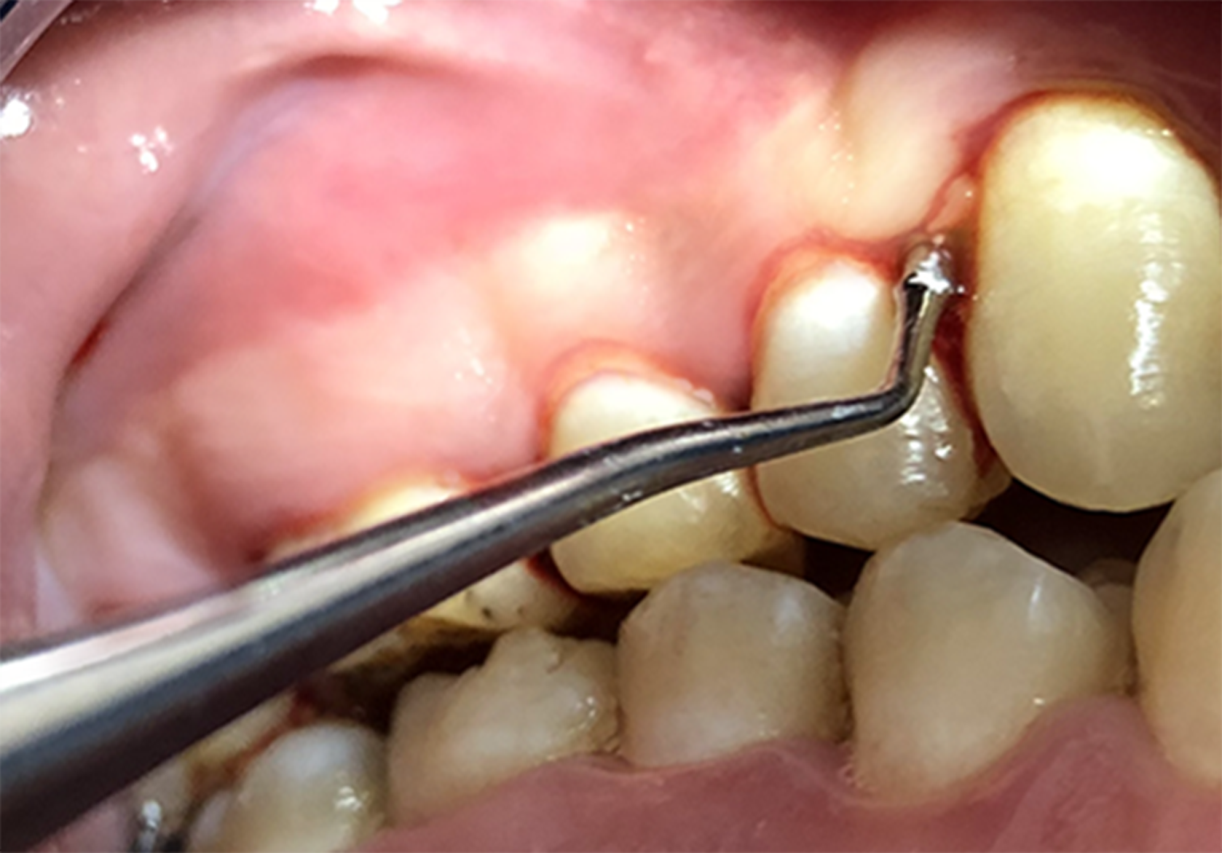


(e)


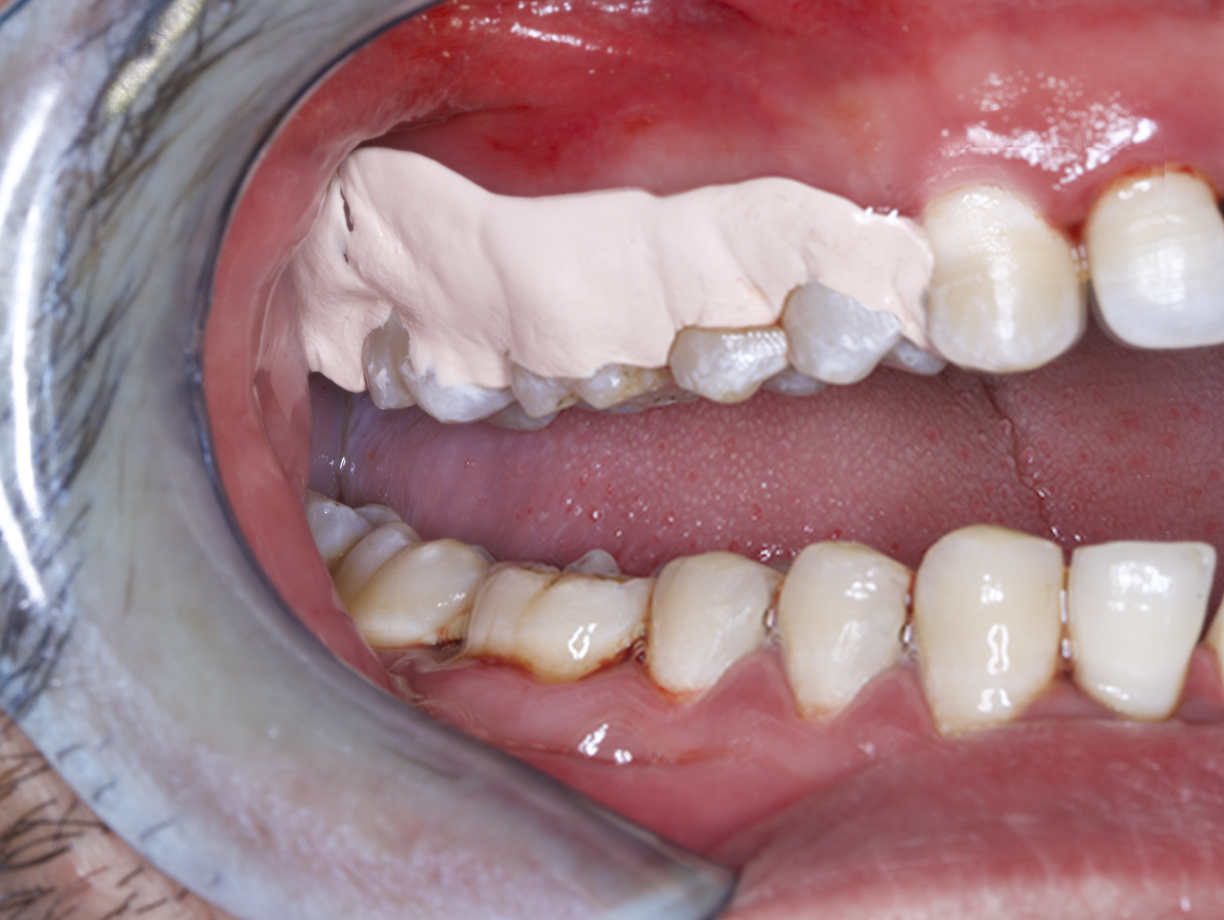


(f)

**Figure (3)**


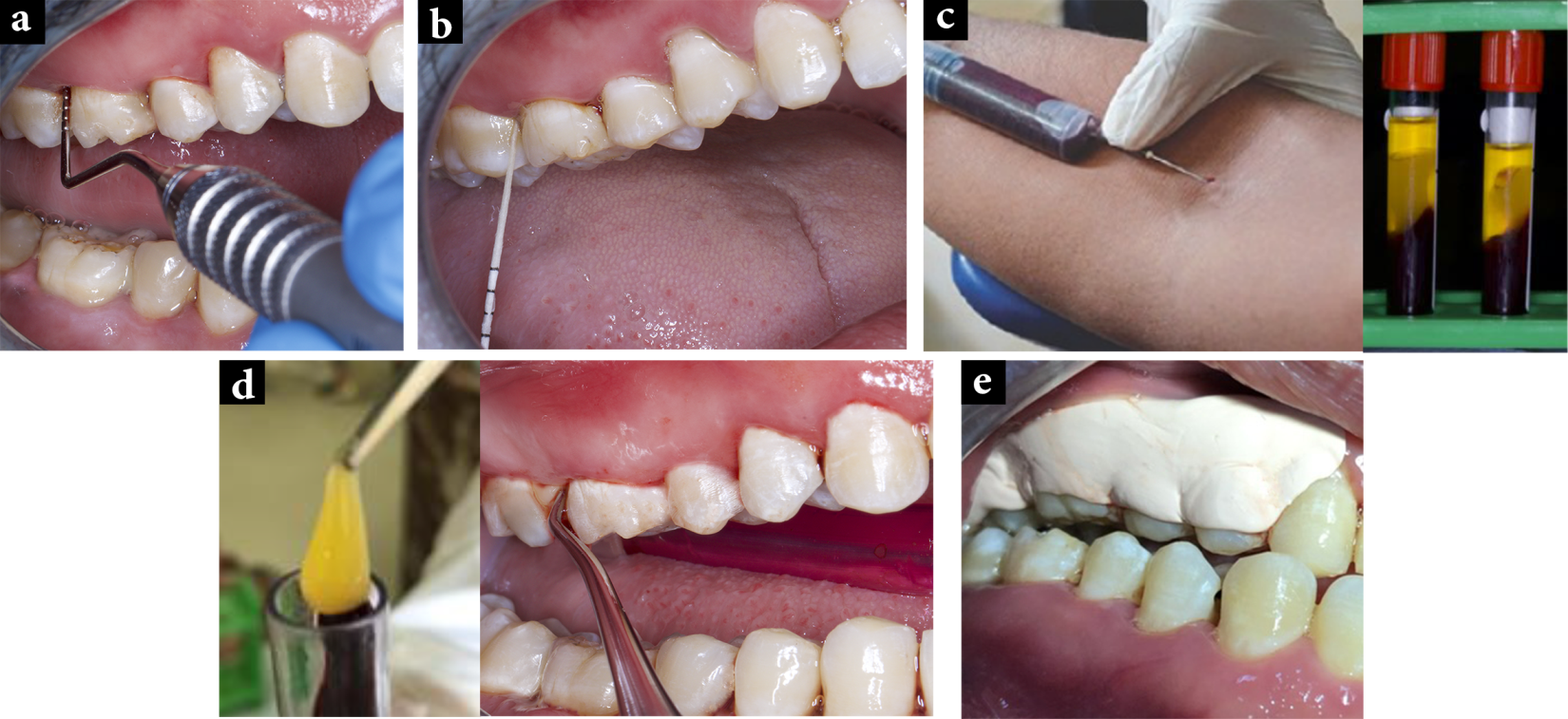


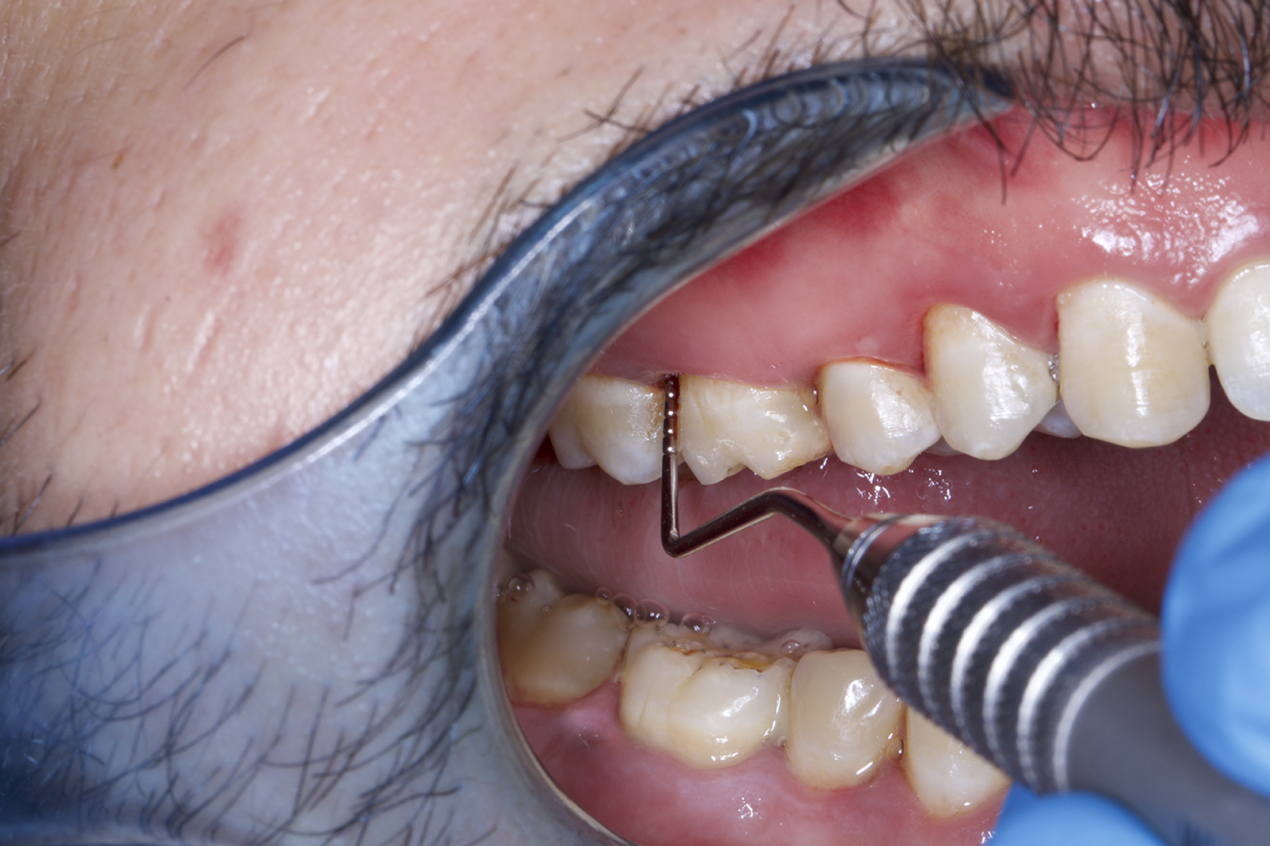


(a)


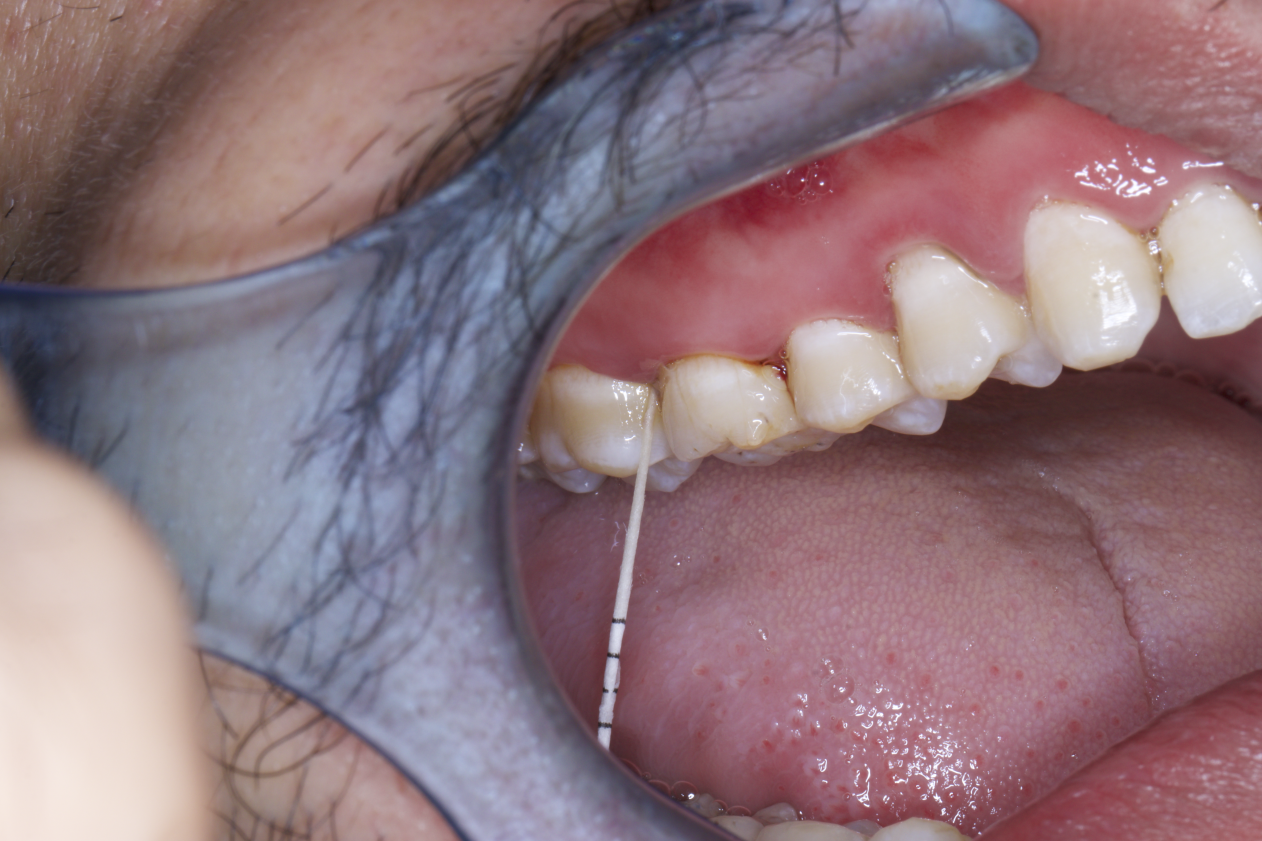


(b)


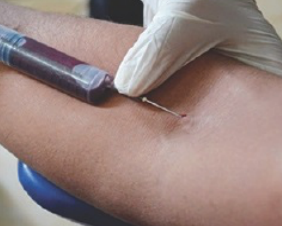

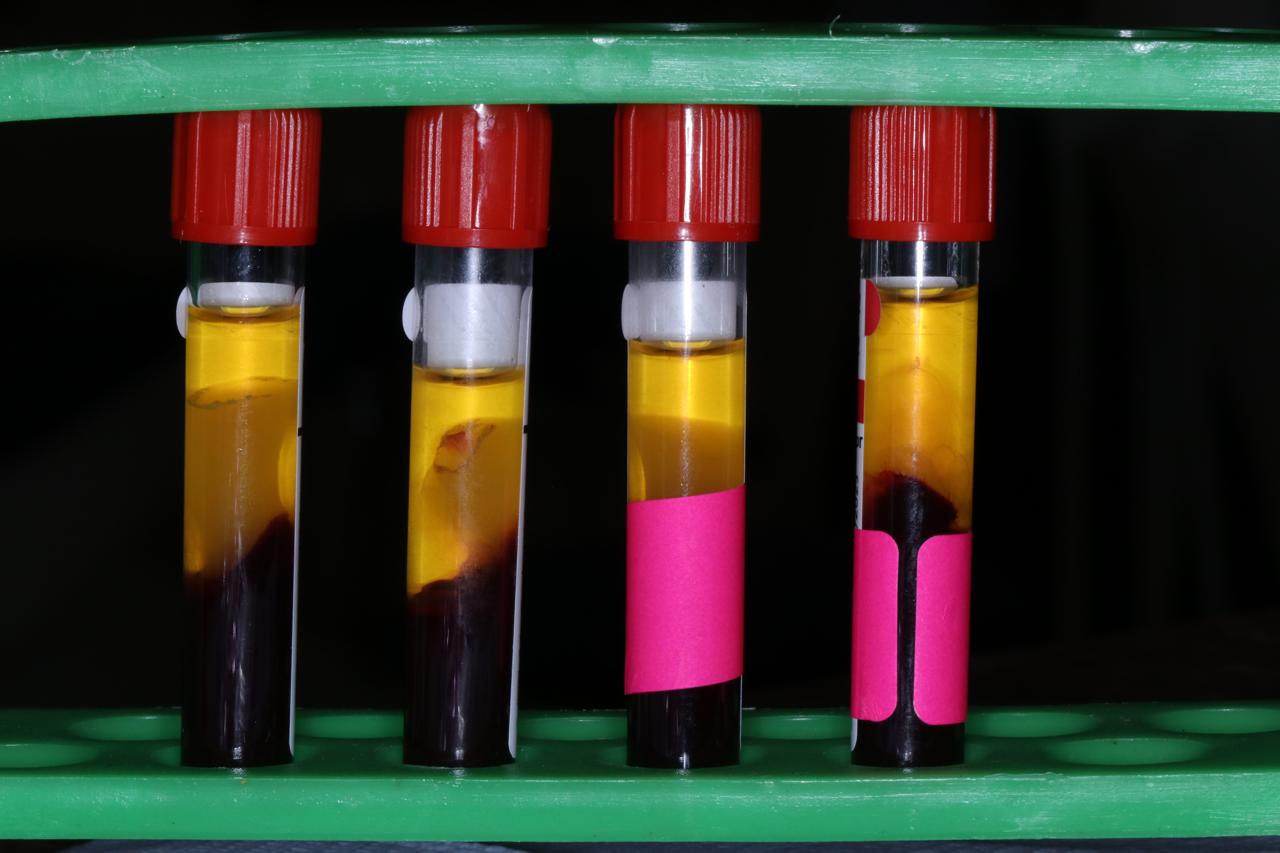


(c)


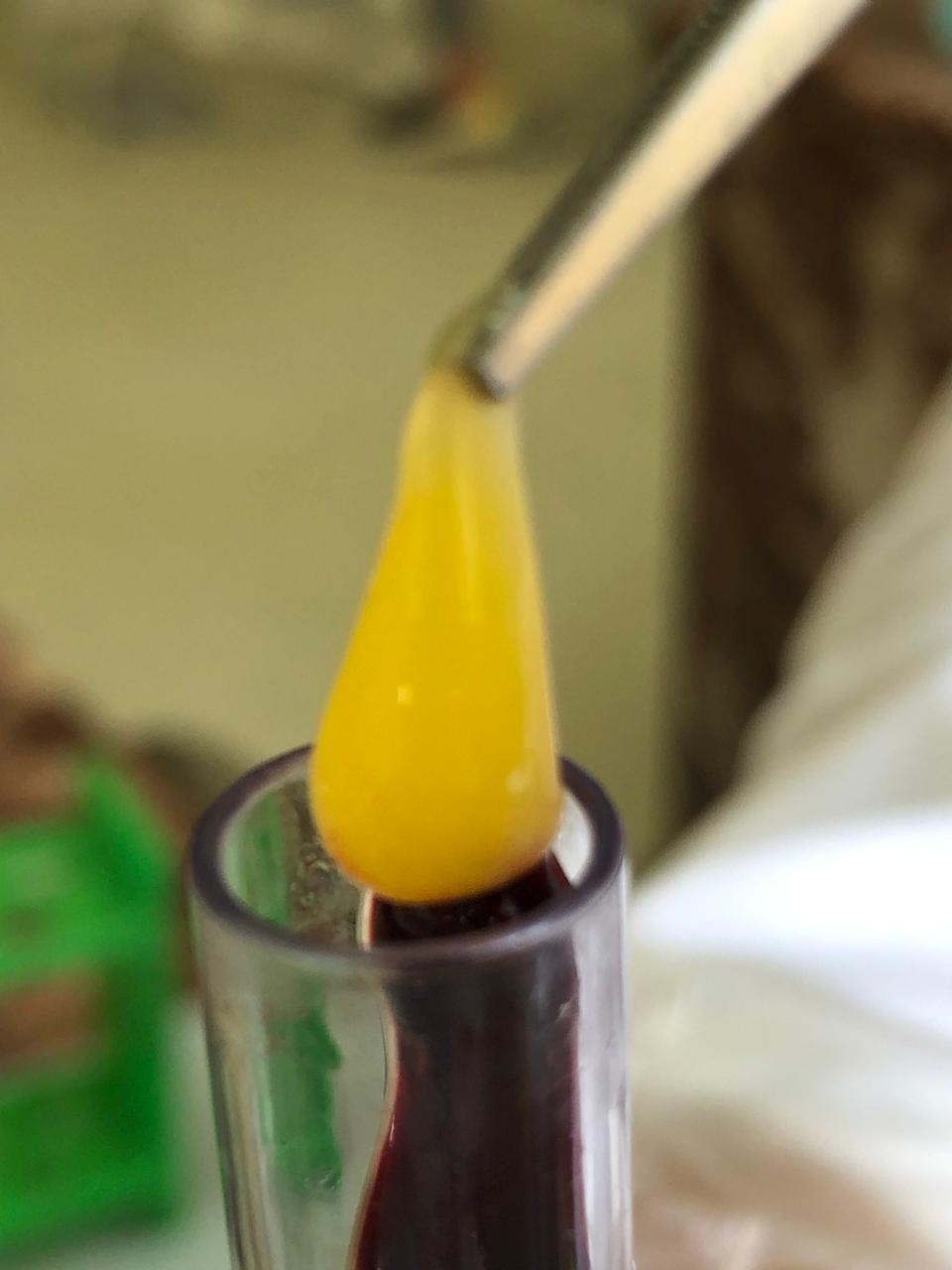


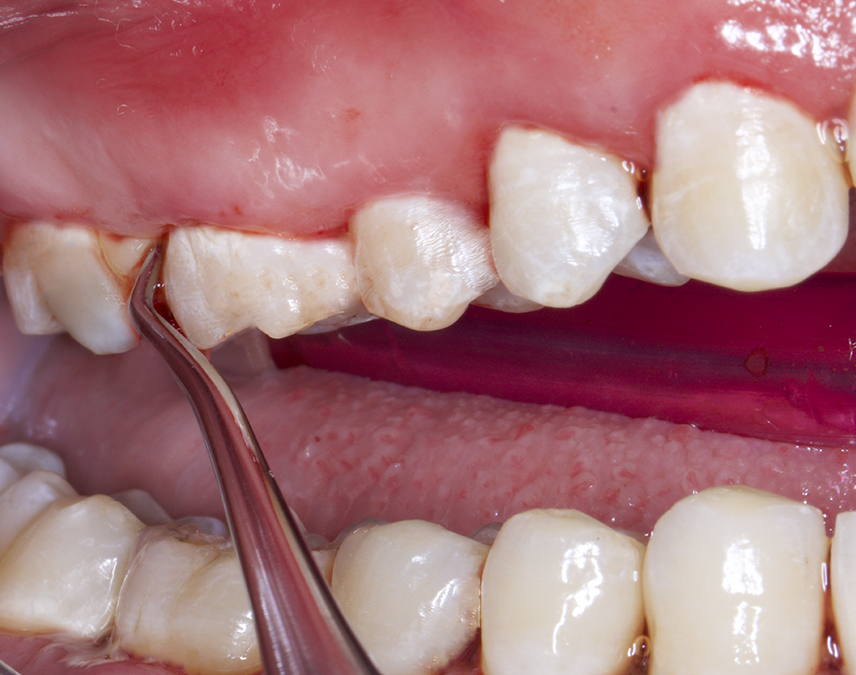


(d)


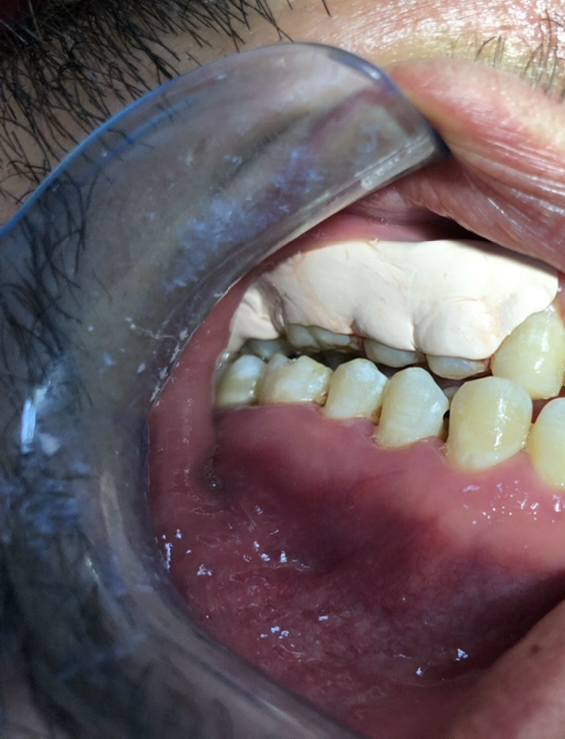


(e)

Supplement: Supplementary file 1 — Supplementary Material 1 [file 12903_2024_5254_MOESM1_ESM.docx]

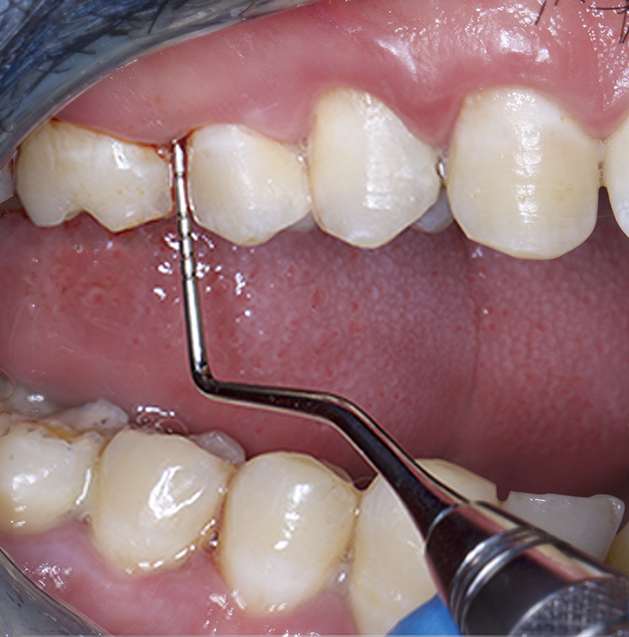

Supplement: Supplementary file 2 — Supplementary Material 2 [file 12903_2024_5254_MOESM2_ESM.tif]

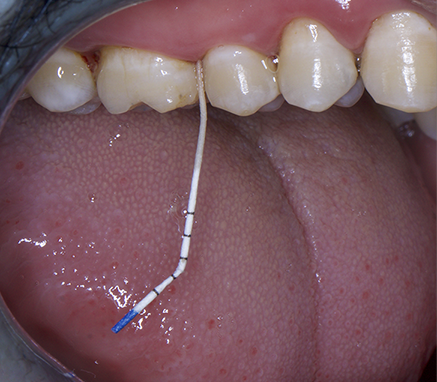

Supplement: Supplementary file 3 — Supplementary Material 3 [file 12903_2024_5254_MOESM3_ESM.tif]

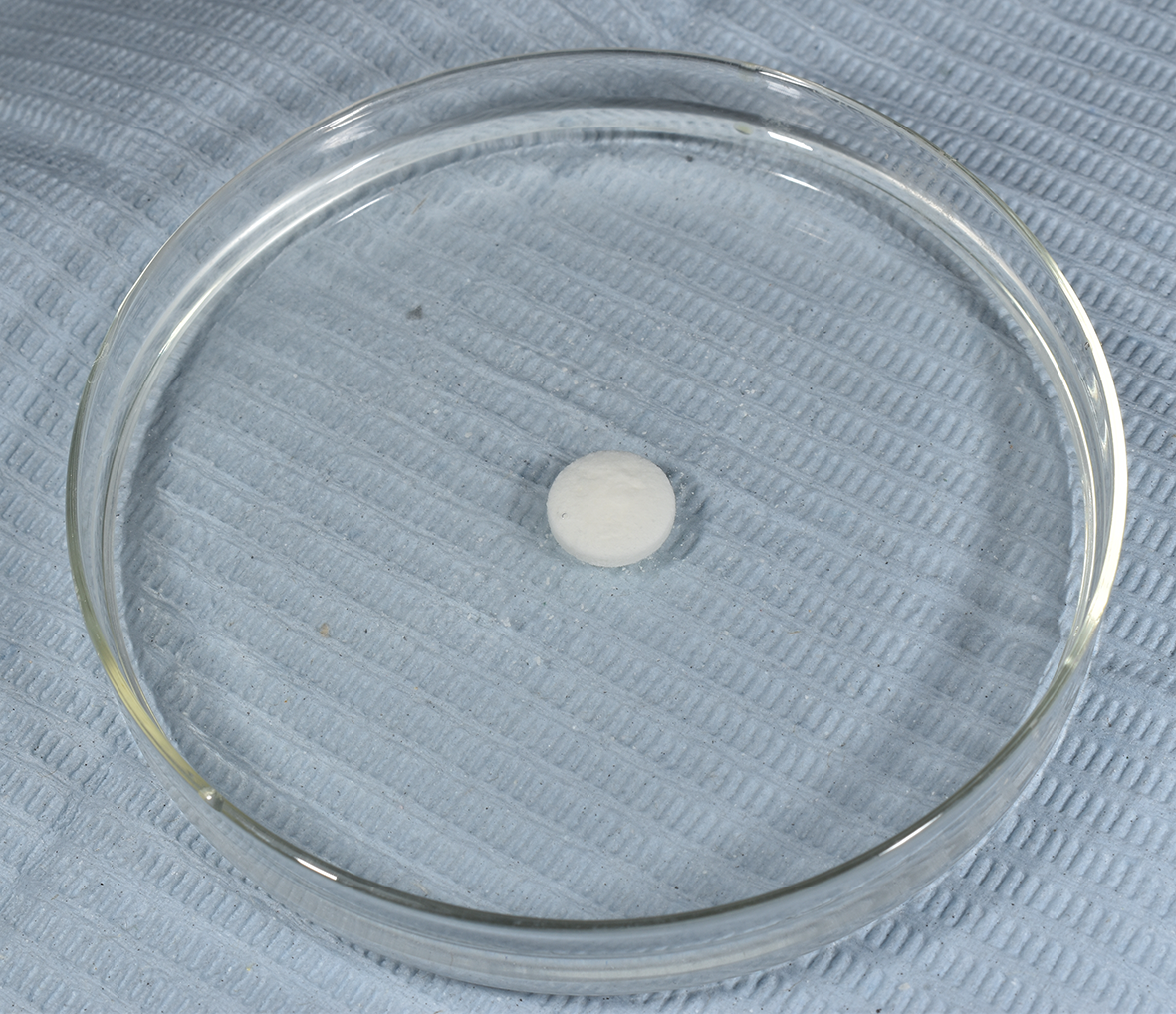

Supplement: Supplementary file 4 — Supplementary Material 4 [file 12903_2024_5254_MOESM4_ESM.tif]

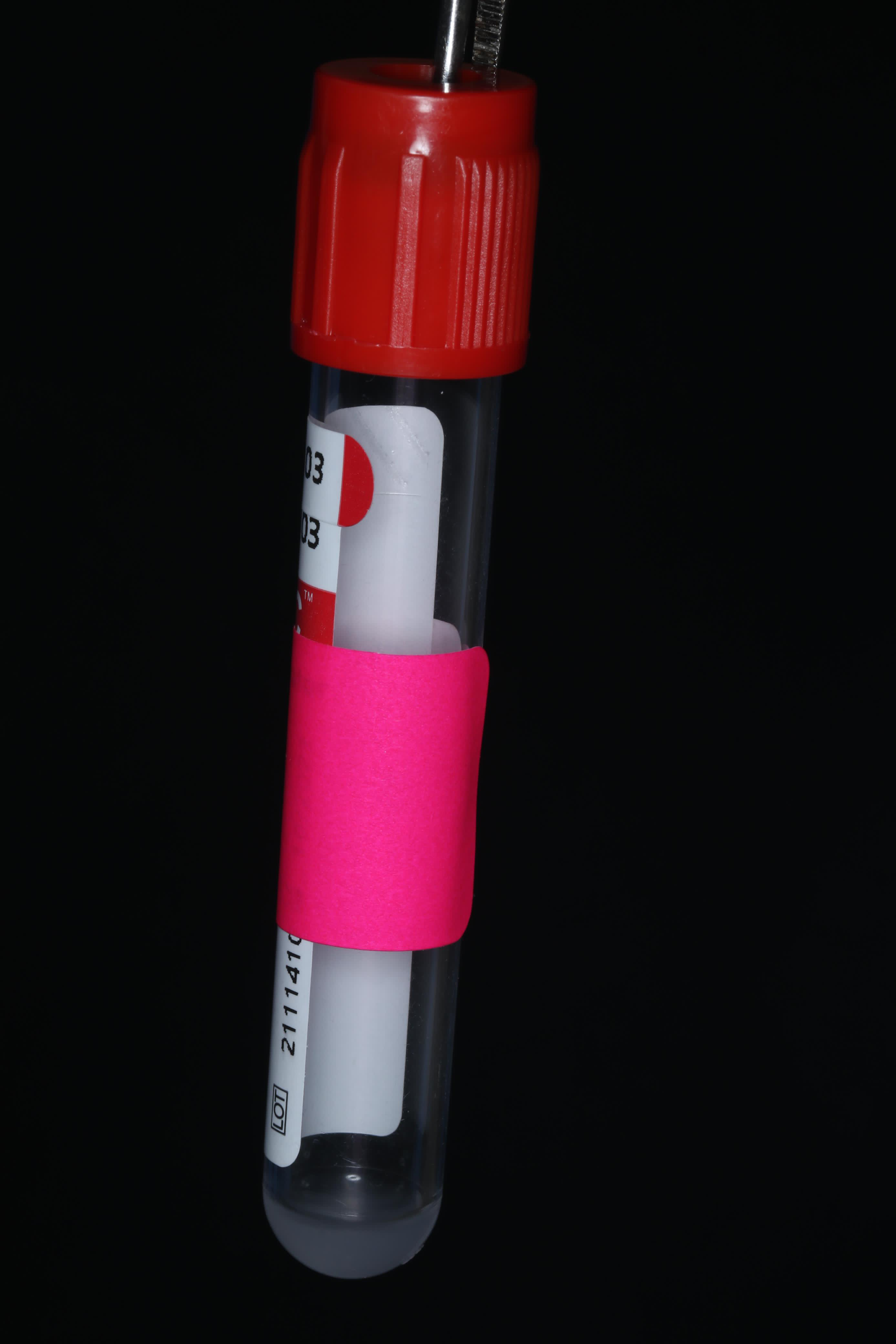

Supplement: Supplementary file 5 — Supplementary Material 5 [file 12903_2024_5254_MOESM5_ESM.tif]

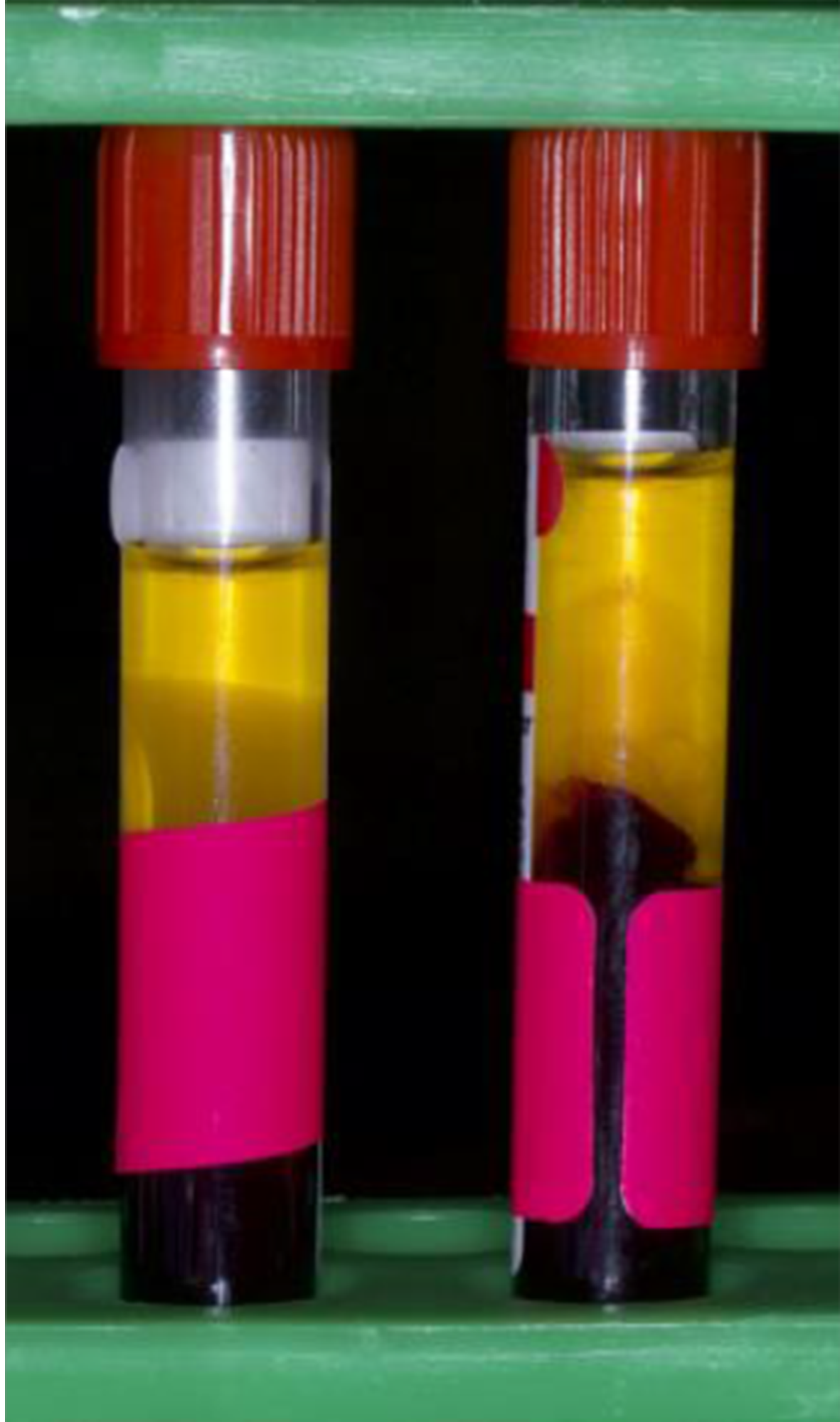

Supplement: Supplementary file 6 — Supplementary Material 6 [file 12903_2024_5254_MOESM6_ESM.tif]

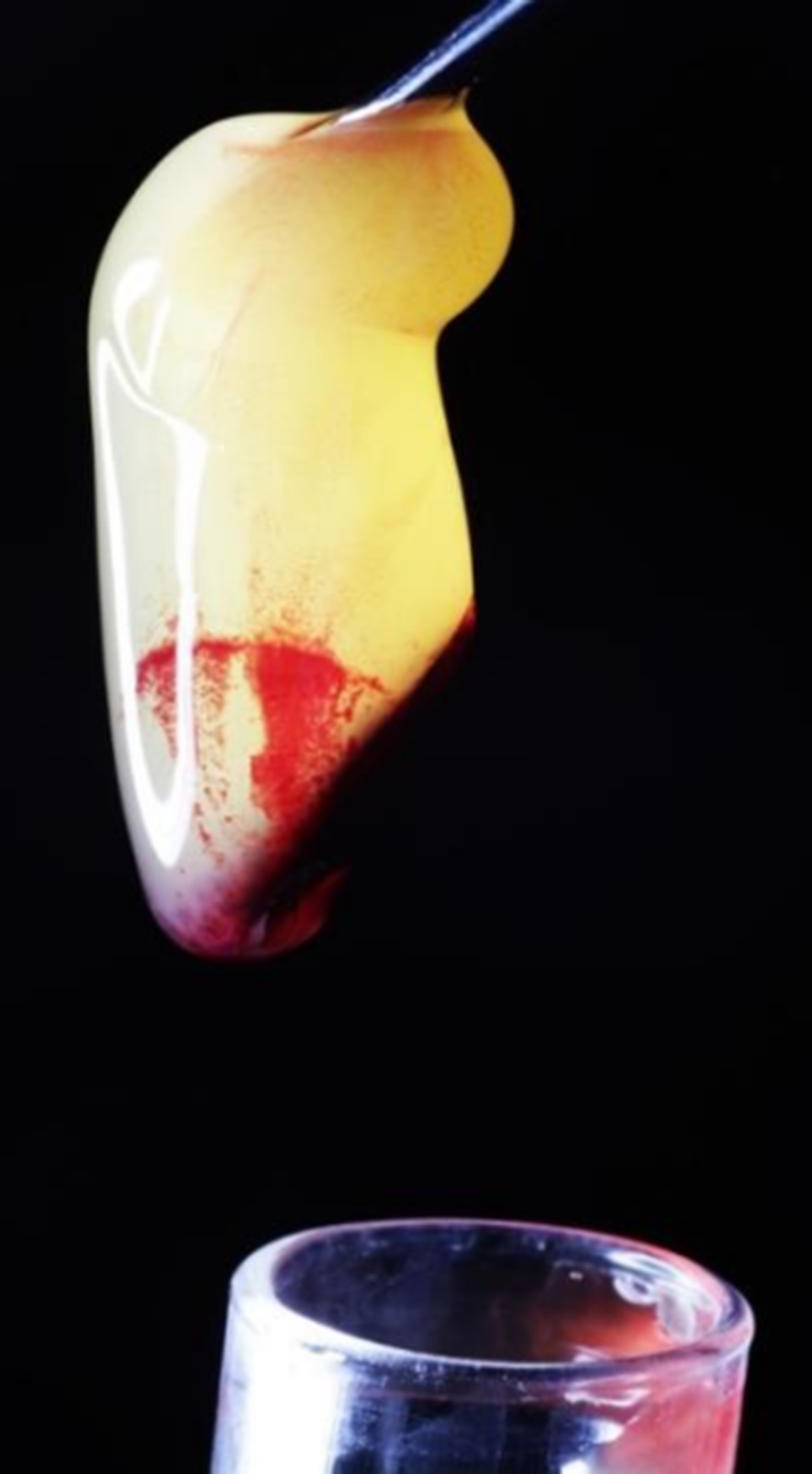

Supplement: Supplementary file 7 — Supplementary Material 7 [file 12903_2024_5254_MOESM7_ESM.tif]

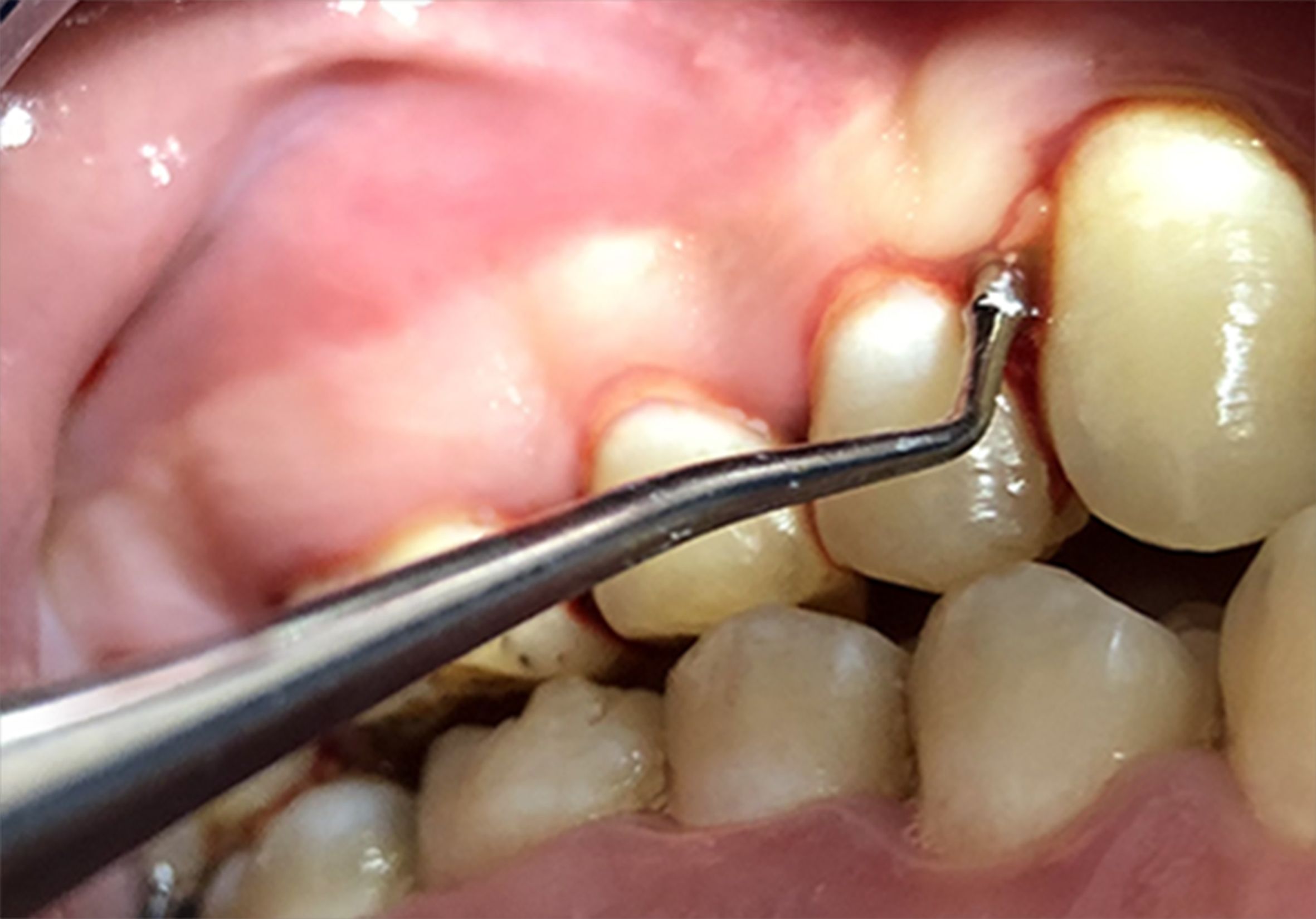

Supplement: Supplementary file 8 — Supplementary Material 8 [file 12903_2024_5254_MOESM8_ESM.tif]

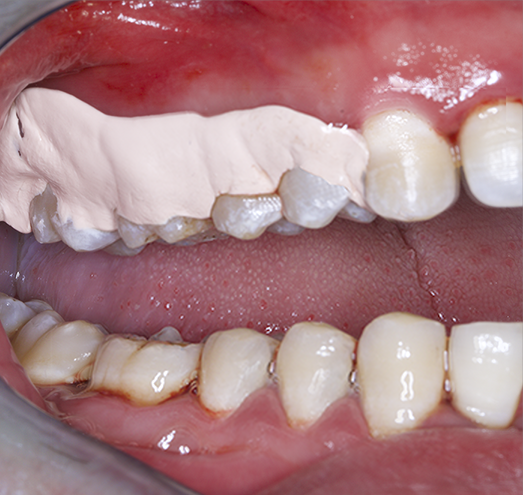

Supplement: Supplementary file 9 — Supplementary Material 9 [file 12903_2024_5254_MOESM9_ESM.tif]

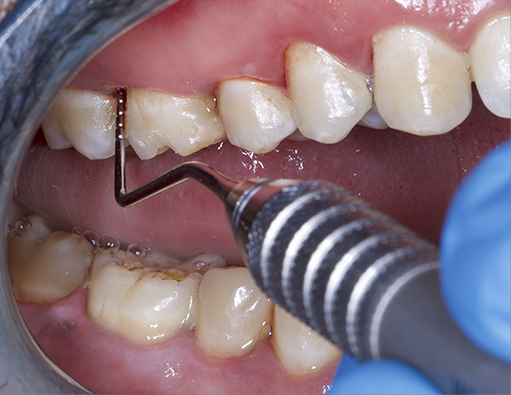

Supplement: Supplementary file 10 — Supplementary Material 10 [file 12903_2024_5254_MOESM10_ESM.tif]

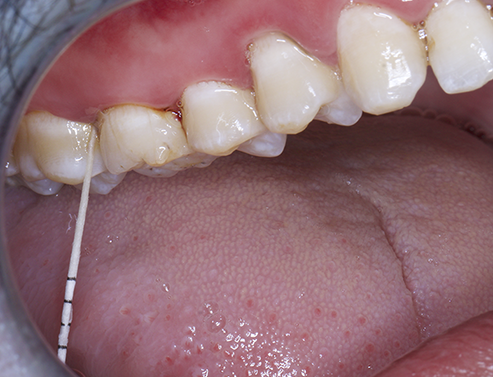

Supplement: Supplementary file 11 — Supplementary Material 11 [file 12903_2024_5254_MOESM11_ESM.tif]

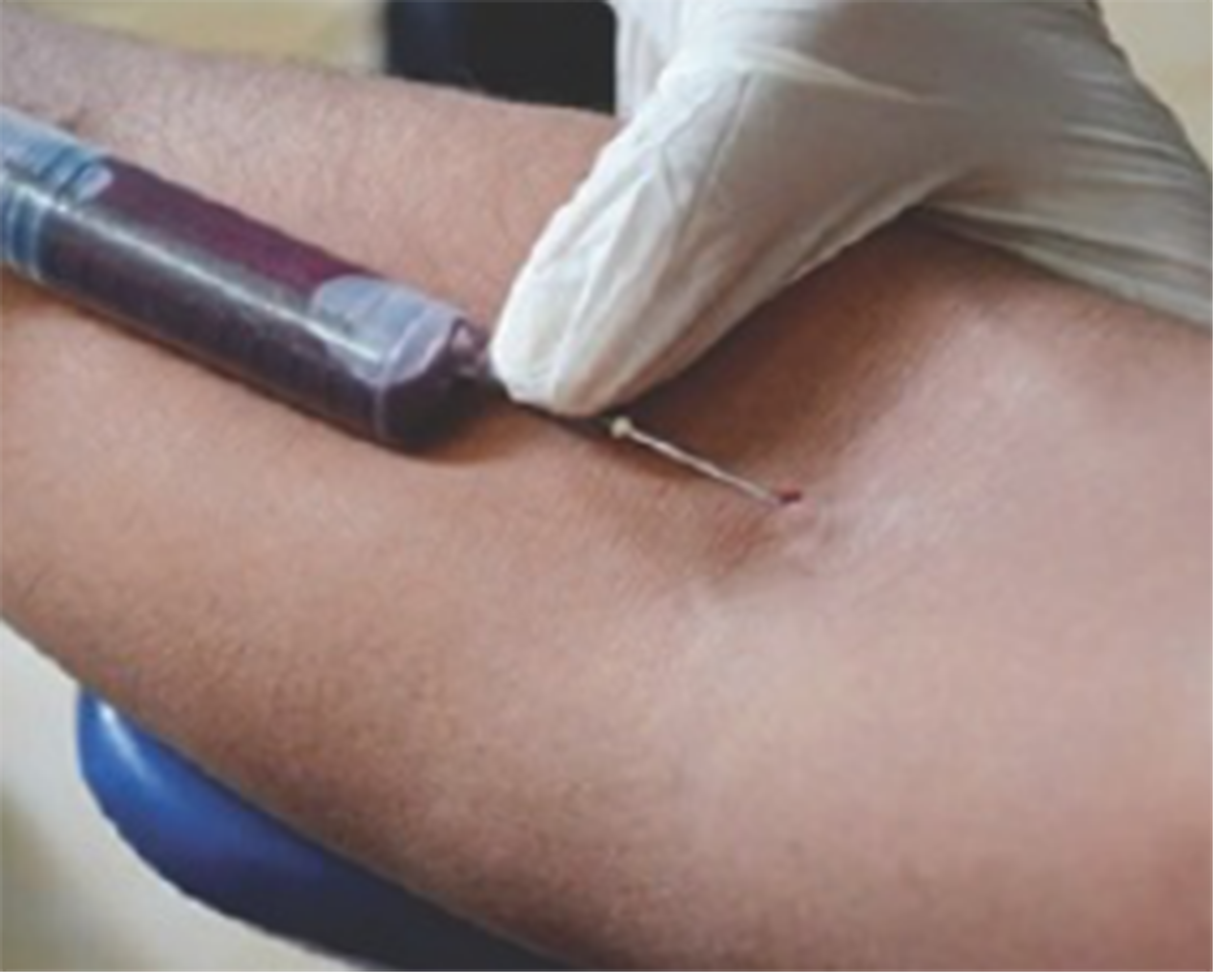

Supplement: Supplementary file 12 — Supplementary Material 12 [file 12903_2024_5254_MOESM12_ESM.tif]

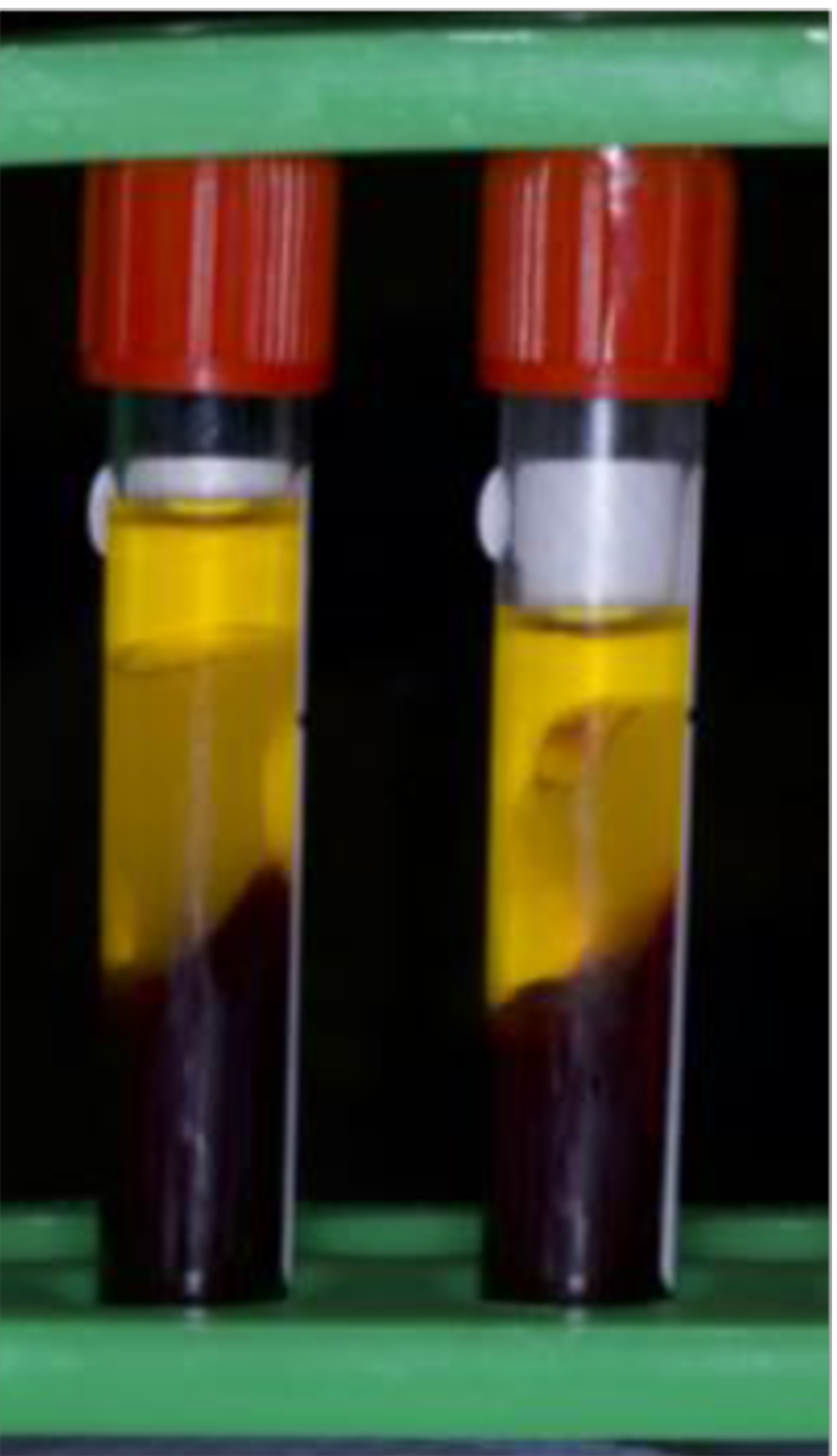

Supplement: Supplementary file 13 — Supplementary Material 13 [file 12903_2024_5254_MOESM13_ESM.tif]

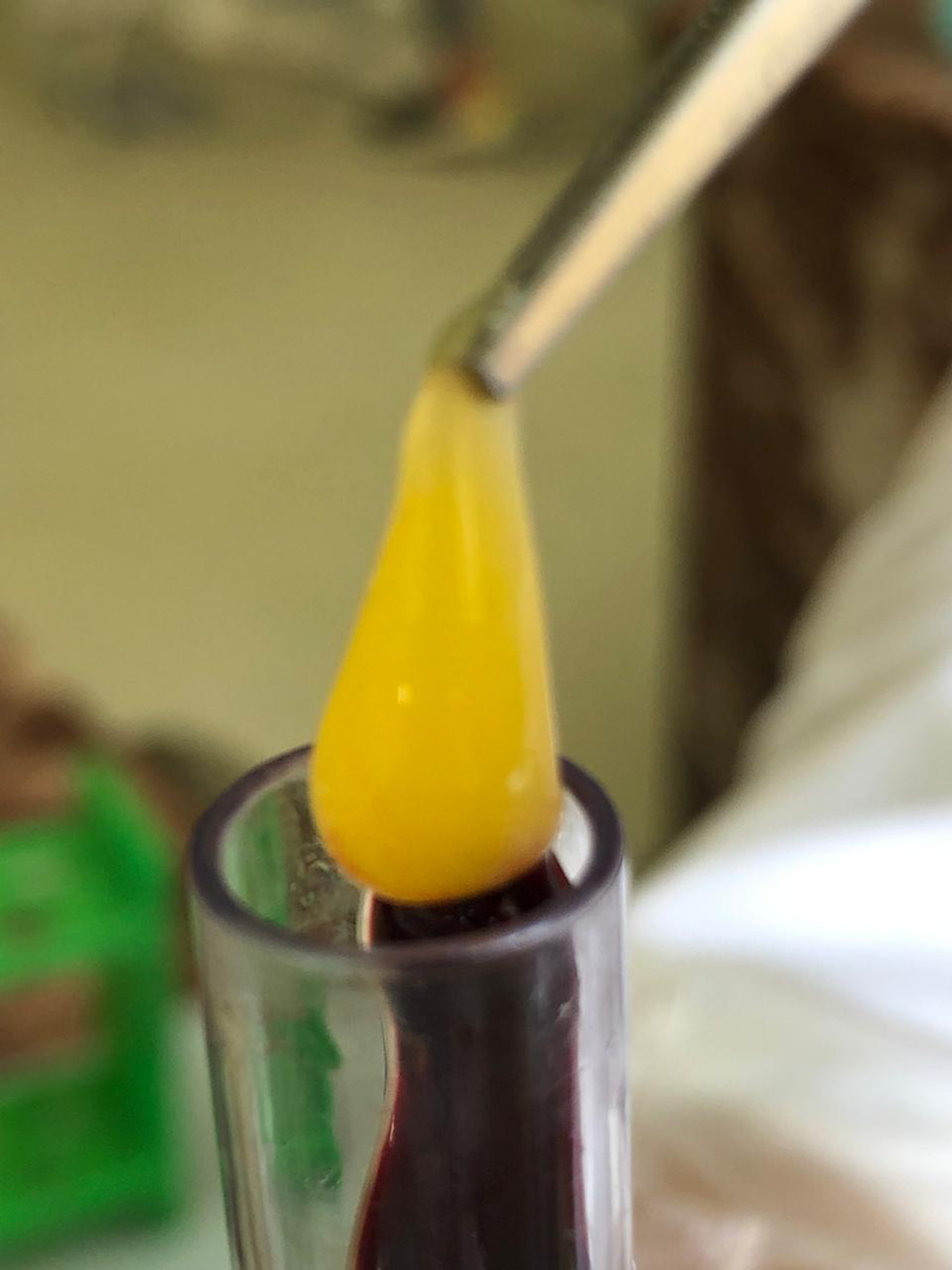

Supplement: Supplementary file 14 — Supplementary Material 14 [file 12903_2024_5254_MOESM14_ESM.tif]

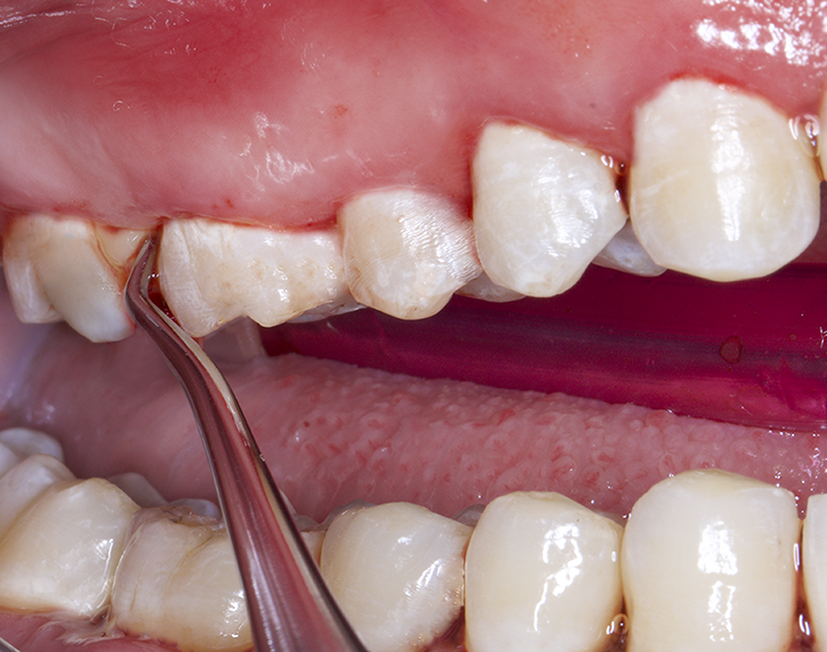

Supplement: Supplementary file 15 — Supplementary Material 15 [file 12903_2024_5254_MOESM15_ESM.tif]

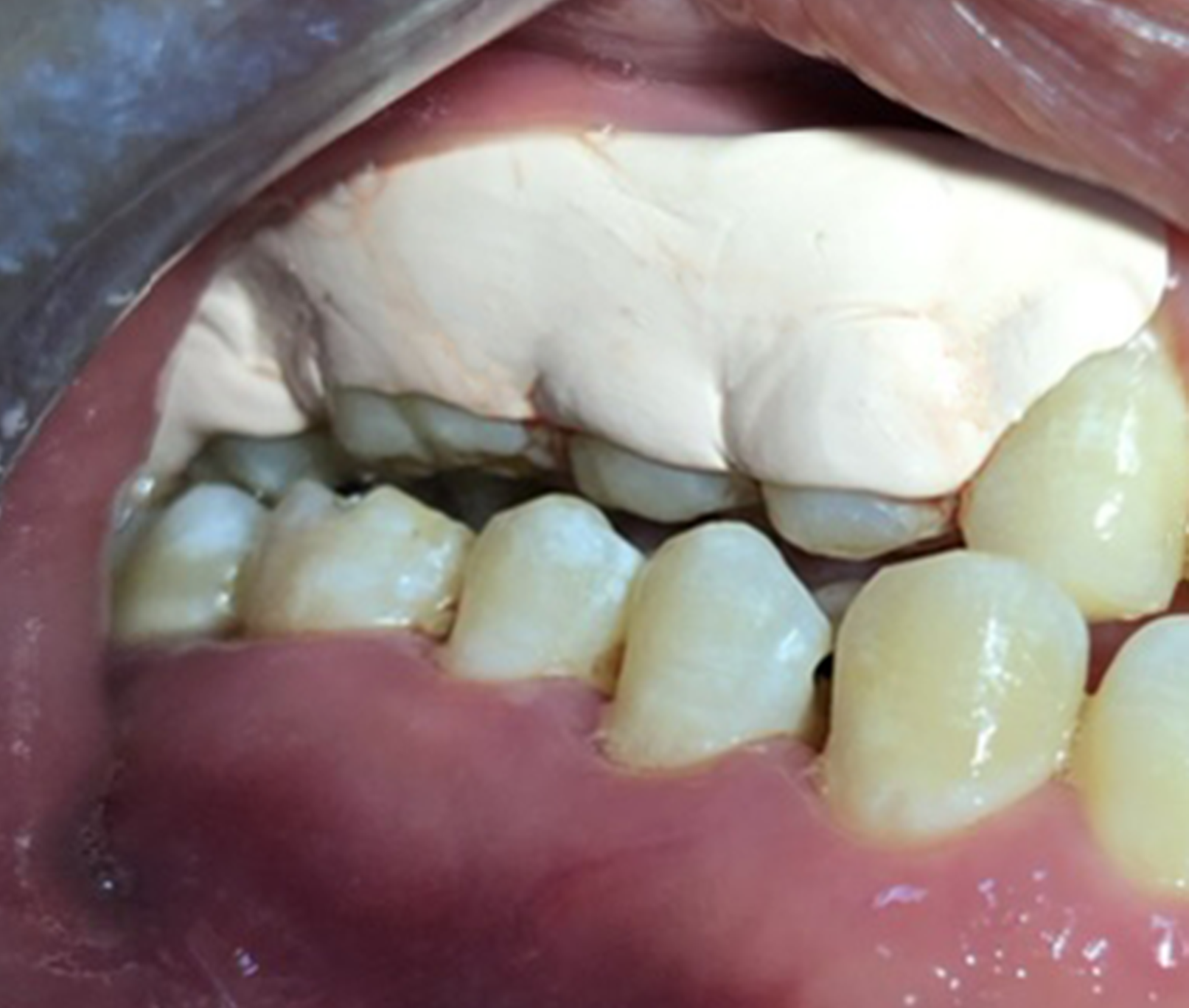

Supplement: Supplementary file 16 — Supplementary Material 16 [file 12903_2024_5254_MOESM16_ESM.tif]
